# An exploration of mortality risk factors in non-severe pneumonia in children using clinical data from Kenya

**DOI:** 10.1186/s12916-017-0963-9

**Published:** 2017-11-13

**Authors:** Timothy Tuti, Ambrose Agweyu, Paul Mwaniki, Niels Peek, Mike English, on behalf of the Clinical Information Network Author Group

**Affiliations:** 1 KEMRI – Wellcome Trust Research Programme, Nairobi, Kenya; 2 Centre for Health Informatics, Division of Informatics, Imaging & Data Sciences, Faculty of Biology, Medicine and Health, The University of Manchester, Manchester Academic Health Science Centre, Manchester, UK; 3 NIHR Greater Manchester Primary Care Patient Safety Translational Research Centre, Manchester, UK; 4 Nuffield Department of Medicine, Oxford University, Oxford, UK

**Keywords:** Pneumonia, Risk factors, Guidelines, Pediatrics, Machine learning, Decision support techniques

## Abstract

**Background:**

Childhood pneumonia is the leading infectious cause of mortality in children younger than 5 years old. Recent updates to World Health Organization pneumonia guidelines recommend outpatient care for a population of children previously classified as high risk. This revision has been challenged by policymakers in Africa, where mortality related to pneumonia is higher than in other regions and often complicated by comorbidities.

This study aimed to identify factors that best discriminate inpatient mortality risk in non-severe pneumonia and explore whether these factors offer any added benefit over the current criteria used to identify children with pneumonia requiring inpatient care.

**Methods:**

We undertook a retrospective cohort study of children aged 2–59 months admitted with a clinical diagnosis of pneumonia at 14 public hospitals in Kenya between February 2014 and February 2016. Using machine learning techniques, we analysed whether clinical characteristics and common comorbidities increased the risk of inpatient mortality for non-severe pneumonia. The topmost risk factors were subjected to decision curve analysis to explore if using them as admission criteria had any net benefit above the current criteria.

**Results:**

Out of 16,162 children admitted with pneumonia during the study period, 10,687 were eligible for subsequent analysis. Inpatient mortality within this non-severe group was 252/10,687 (2.36%). Models demonstrated moderately good performance; the partial least squares discriminant analysis model had higher sensitivity for predicting mortality in comparison to logistic regression.

Elevated respiratory rate (≥70 bpm), age 2–11 months and weight-for-age Z-score (WAZ) < –3SD were highly discriminative of mortality. These factors ranked consistently across the different models. For a risk threshold probability of 7–14%, there is a net benefit to admitting the patient sub-populations with these features as additional criteria alongside those currently used to classify severe pneumonia. Of the population studied, 70.54% met at least one of these criteria. Sensitivity analyses indicated that the overall results were not significantly affected by variations in pneumonia severity classification criteria.

**Conclusions:**

Children with non-severe pneumonia aged 2–11 months or with respiratory rate ≥ 70 bpm or very low WAZ experience risks of inpatient mortality comparable to severe pneumonia. Inpatient care is warranted in these high-risk groups of children.

**Electronic supplementary material:**

The online version of this article (doi:10.1186/s12916-017-0963-9) contains supplementary material, which is available to authorized users.

## Background

Pneumonia is the leading infectious cause of mortality in children aged less than 5 years, accounting for almost one million deaths each year worldwide. It is estimated that more than half the burden is experienced in sub-Saharan Africa (SSA) [1], where there are limited disease surveillance and research and weak health systems [2]. Based on severity, 7–13% of these cases are considered to be life-threatening and require hospitalisation [3].

The World Health Organization (WHO) has recommended the use of simple algorithm-based clinical guidelines to guide diagnosis and treatment of pneumonia for more than three decades [4]. The revised WHO pneumonia classification [5] (Additional file 1) considers the former “non-severe” and “severe” pneumonia categories as a single group now referred to as “pneumonia” for whom outpatient care is now recommended. There has been reluctance to adopt this new classification, particularly in SSA, where policymakers have raised concerns that children with pneumonia manifesting with lower chest wall indrawing represent a population with a high risk of death [6, 7]. Observational data from various studies identify clinical characteristics that occur commonly as comorbidities among children with pneumonia in SSA such as malaria, diarrhoea/dehydration and anaemia [8, 9]. Yet the current WHO guidelines fail to include the presence of these risk factors for classifying severity. The exploration of if and how risk profiles vary among children with non-severe pneumonia under the new guidelines has yet to be demonstrated. Previous studies describing risk factors for pneumonia mortality have included populations with very low coverage of the conjugate vaccines against *Streptococcus pneumoniae* and *Haemophilus influenzae* type B (the leading causes of bacterial pneumonia), and the analyses reported have had limited application for clinical decision making.

The aim of this study was to identify demographic and clinical factors that best discriminate risk of death among children with non-severe pneumonia as defined by WHO clinical criteria, clinician diagnosis and penicillin monotherapy treatment using robust modelling approaches. Additionally, we were interested in application of decision-analytic approaches to model outputs. To this end, the identified factors would then be evaluated on whether they offer any added benefit over the current severity criteria used to determine pneumonia patients to admit.

## Methods

### Reporting

The reporting of this observational study follows the Strengthening of reporting of observational studies in epidemiology (STROBE) statement [10], which is a set of recommendations for the reporting of observational studies in epidemiology (cohort, case-control studies and cross-sectional studies) [10].

### Ethics, consent and permissions

This study was approved by the Scientific and Ethics Review Unit of the Kenya Medical Research Institute (KEMRI). Additionally, it was approved by the Ministry of Health, with the Medical Superintendents of participant hospitals giving consent for participation. Individual consent for access to de-identified patient data was not required.

### Study design and setting

This study was a retrospective cohort study at 14 public hospitals in Kenya with each having at least 1000 annual paediatric admissions, purposefully selected to represent two main regional groupings based on high or low malaria prevalence. The study was embedded within a collaborative health information network developed to help improve outcomes of care, accelerate knowledge discovery and advance cross-domain development of digital architecture in support of research in a low-income setting. This Clinical Information Network (CIN) is described in detail elsewhere [11].

### Study participants, data sources and management

All paediatric inpatient children admitted to the selected hospitals from 1 February 2014 to 28 February 2016, aged 2–59 months who had non-severe pneumonia at admission were eligible for inclusion in this study. This was determined from clinician diagnosis and clinical signs documented in patient records. To avoid confusion, we have used the term pneumonia to refer to children with a documented clinical diagnosis of pneumonia, and the terms non-severe and severe pneumonia to refer to those for whom WHO, under the 2013 revised definitions, recommends outpatient and inpatient care, respectively. The ideally diagnosed and managed population consisted of patients with a clinician-assigned admission diagnosis of non-severe pneumonia, who, as based on WHO guidelines, had clinical signs supporting this diagnosis and were treated with penicillin monotherapy. We excluded children born before the introduction of the pneumococcal conjugate vaccine to the national childhood immunisation schedule in January 2011; thus, the study population included children who were born after the introduction of both the pneumococcal and *Haemophilus influenzae* type B (Hib) conjugate vaccines (introduced nationally in 2001). Comprehensive data collected for these admissions comprised clinical, investigation and treatment data focused on admission and discharge events, with up to 350 variables per patient encounter collected. These variables span different disease conditions. A detailed description of the methods of data collection and analysis is reported elsewhere [11]. For our study, 37 variables were used: 18 variables were used in pneumonia classification criteria and in identifying the analysis population, and 19 variables were used for subsequent statistical analysis — 6 of which were interaction terms (variables used to test whether the effect of one independent variable differs depending on the level of another independent variable). In brief, hospitals implemented two data collection tools (a paediatric admission record and a discharge form) with one clerical assistant posted to each hospital to collect data from the medical records and laboratory reports. Data collection was conducted as soon as possible after discharge through abstracting data from inpatient paper records into a non-propriety electronic tool, Research Electronic Data Capture (REDCap) [12]. Data quality reports were generated by R scripts [13] based on validation rules and metadata pulled from REDCap’s application programming interface. These reports were fed back to the hospitals to improve the quality of clinical data used in this research. We have reported in detail elsewhere the process by which we established a clinical information network in Kenya, the multiple unique challenges we faced including the development of new data collection procedures and new methods to implement the provision of accurate reporting to hospitals [11].

### Quantitative variables

Our prognostic models focused on paediatric inpatient hospital mortality, described by a binary variable (dead or alive). Predictors were identified a priori guided by clinical expert opinion and literature review. We selected variables posited to be associated with mortality and which could also be widely ascertained in low-resource clinical settings. To denote nutritional status, we used recorded weight and age to retrospectively compute weight-for-age Z-scores (WAZ) using WHO child growth standards [14], as data for these two variables were complete for the majority of patients studied. This resulted in the following predictors being selected, covering demographics and clinical characteristics: *Age < 12 months (binary), Sex-Female (binary), Respiratory rate ≥ 70 breaths/min (binary), Temperature ≥ 39 °C (binary), Weight-for-age Z-score (ordinal* — *3 levels), Dehydration status (ordinal* — *3 levels), Pallor (ordinal* — *3 levels), Malaria status (ordinal* — *3 levels), Presence of ≥ 1 comorbidity (binary), Hospital in malaria endemic area (binary), Acute nutrition status (binary).* Table 1 provides description of levels of the ordinal variables. Severity of pneumonia was categorised based on documented WHO clinical criteria (non-severe vs severe) [15]. Dummy binary variables were created for all levels in ordinal predictors. All predictors were assessed at the time of admission.

**Table 1:** Descriptive summary statistics of the included predictors and variables of interest (*N* = 10,687)

Indicator	Levels	Number, *N* (%)
Age < 12 months	No	5719 (53.51%)
	Yes	4968 (46.49%)
Female	No	5856 (54.8%)
	Yes	4736 (44.32%)
	Missing	95 (0.89%)
Pallor	None	7613 (71.24%)
	Mild/moderate	374 (3.5%)
	Severe	98 (0.92%)
	Missing	2602 (24.35%)
Respiratory rate ≥ 70 breaths/min	No	6622 (61.96%)
	Yes	4065 (38.04%)
Weight-forage Z-score (WAZ)	> –2SD	8311 (77.77%)
	–2 to –3SD	1202 (11.25%)
	< –3SD	719 (6.73%)
	Missing	455 (4.26%)
Temperature ≥ 39 °C	No	6577 (61.54%)
	Yes	1257 (11.76%)
	Missing	2853 (26.7%)
Dehydration	No dehydration	10,026 (93.81%)
	Some dehydration	622 (5.82%)
	Missing	39 (0.36%)
Malaria	No malaria	9611 (89.93%)
	Non-severe malaria	1076 (10.07%)
Hospital located in malaria endemic area	Yes	4447 (41.61%)
	No	6240 (58.39%)
Acute malnutrition	None/at risk	10,572 (98.92%)
	Moderate	115 (1.08%)
Presence of comorbidity^a^	No	7330 (68.59%)
	Yes	3357 (31.41%)

^a^Admission diagnosis of malaria, diarrhoea, dehydration and anaemia considered

Patients with non-severe pneumonia with a diagnosis of either (1) severe dehydration or (2) severe malaria were recoded to severe pneumonia, since either of these diagnoses would render the respective patients’ ineligible for outpatient care. All cases of severe pneumonia were excluded from the analysis. Pneumonia cases with additional admission diagnosis of meningitis, acute malnutrition and shock were also excluded from the study sample; these conditions follow alternative management protocols under the clinical guidelines [16].

### Statistical methods

Data manipulation and statistical analyses were performed using R software [13] employing the caret package [17]. Categorical data were tabulated and summarised as proportions, while continuous variables were reported with medians and interquartile ranges as appropriate. To evaluate differences in the risk profile between the two groups of inpatient mortality outcomes, and identify predictors that substantively account for these differences, an adjusted multivariable logistic regression model was used.

In previous research, logistic regression modelling has been used to look at risk factors in pneumonia (Ambrose Agweyu, et al., Appropriateness of clinical severity classification of new World Health Organization (WHO) childhood pneumonia guidance: a multi-hospital retrospective cohort study. *The Lancet Global Health*, under review). However, due to the violation of assumptions of independence of predictors (e.g. *age* variable would be collinear with *Weight-for-age Z-score* variable, etc.) and the limited understanding of the relationship of the predictors with mortality in non-severe pneumonia, this approach is susceptible to incorrect inferences about relationships between explanatory and response variables. Additionally, apart from model coefficients and significance tests, logistic regression models offer limited guidance on feature selection from model outputs that can guide future intervention design. Feature selection is defined and explained further in Additional file 2: Table S1 and in published reports [18, 19]. Therefore, the magnitude of coefficients included in the traditional adjusted logistic regression models might not be good indicators of clinical value of features, since they do not incorporate clinical consequences involved in targeting those features [20, 21].

To address these challenges in generating decision-analytic solutions from logistic models, machine learning techniques were used. These techniques were also explored to test whether, given the available data, models using complex adaptive techniques perform better in determining the inpatient mortality risk associated with non-severe pneumonia given the choice of predictors. The use of these techniques would also provide implicit feature selection as part of the model output, in addition to allowing us to evaluate whether there was consistency of findings given the different model choice. The machine learning models used in analysis were partial least squares - discriminant analysis (PLS-DA) [22], random forests (RFs) [23], support vector machines (SVMs) [24] and elastic nets [25]. Brief descriptions of these models are given in Additional file 2: Table S2. Detailed descriptions of the models are provided in the referenced works. Here we offer an introduction to the techniques used, which may be less familiar.

Model validation was checked by employing a 10-fold internal cross validation on two thirds of the data. The remaining one third of the data was used as the validation set. This is further explained in Additional file 2: Table S1. Variable importance scores, which would guide feature selection, were generated to identify predictor contribution to classification, with higher scores considered more relevant in classification. Detailed explanations of variable importance estimation for the models included in the analysis are reported elsewhere [26]. The selection of critical parameters for each of these modelling techniques was auto-determined by the R caret train function by choosing the tuning parameters that produced the highest values of receiver operating characteristic (ROC) curves where a grid search cross-validation was applied. These parameters are provided in Additional file 2: Table S2.

To evaluate the clinical impact of implementing the models in practice as part of screening algorithms, we performed decision curve analysis, evaluating how different threshold probabilities vary the false-positive and false-negative rate expressed in terms of net benefit [27]. The unit of net benefit is true positives, and the details of its calculation are extensively reported elsewhere [20]. When carrying out a head-to-head comparison of different prediction models on the same population, the interpretation is straightforward — at each clinically relevant probability threshold, the model that has the highest net benefit is preferred. Models are also compared to the extreme choices of designating admitting all and no patients at high risk of inpatient mortality.

#### Learning using imbalanced outcome data

From the description of our outcome (inpatient mortality cases in non-severe pneumonia), we expect the cases to be imbalanced; i.e. the number of positive cases is much smaller than the number of negative cases. This introduces a high possibility of the resulting model being biased towards the dominant class, presenting poor accuracy to classify negative cases. In order to minimise this bias, we used the Synthetic Minority Over-Sampling Technique (SMOTE) filter [28, 29] to address the imbalanced data. The SMOTE technique was used to oversample the negative cases, which eliminated the possibility of information loss. This was achieved by combining the features of existing instances with the features of their nearest neighbours to create additional synthetic instances. More details on this are provided in Additional file 2: Table S3.

#### Missing data

To handle missing data, multiple imputation by chained equations (generating 10 imputed datasets) was performed under a missing at random (MAR) assumption [30].

#### Performance analysis

Model performance was analysed using sensitivity (true positive rate), specificity (true negative rate) and ROC’s area under the curve (AUC). AUC is a combined indicator of sensitivity and specificity, equal to the probability that a classifier will rank a randomly chosen positive instance higher than a randomly chosen negative one [31]. DeLong’s significance test was used to compare the ROC curves from each model type [32].

#### Sensitivity analysis

We used alternative definitions of pneumonia severity to conduct sensitivity analyses. We performed analyses using clinician-defined severity (non-severe vs severe) and choice of initial treatment prescribed to the patient at admission (benzyl penicillin monotherapy vs alternative broad spectrum treatment) against the definition based on WHO severity criteria as the “gold-standard”. The three definitions should ideally represent populations that overlap perfectly; however, inconsistencies have been observed in previous work [33, 34]. Comprehensive comparisons of risk where pneumonia guidelines were not adhered to (which is a common occurrence in low-resource settings) are lacking in the literature. Here, the key consideration was the widely reported lack of concordance of health workers’ pneumonia severity classification practices in comparison to clinical guidelines under routine conditions [33–35].

## Results

**Fig. 1:**
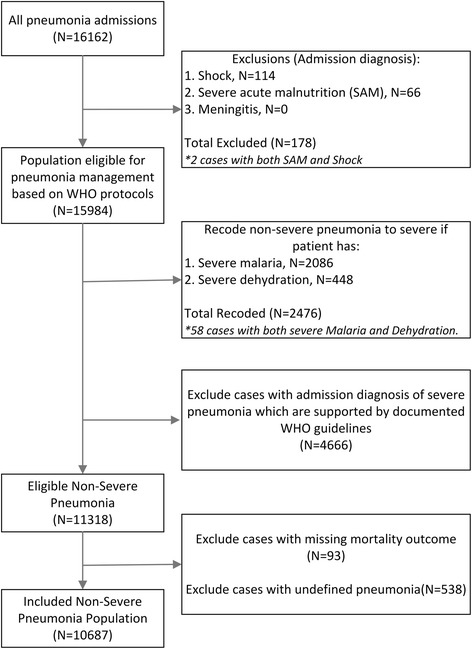
Flow diagram of eligible study participants. The final non-severe pneumonia cases included in subsequent analysis represent the combined number of all non-severe cases as defined by the three classification criteria illustrated in Additional file 3

Figure 1 depicts the study population inclusion process. Out of 16,162 children admitted with pneumonia (severe and non-severe) over the study period, 10,687 cases of non-severe pneumonia were identified according to the 2013 WHO guidelines, clinician diagnosis or penicillin monotherapy treatment. Table 1 below gives characteristics of paediatric patients and information, indicating the number of patients with missing data for the variables of interest. It also gives the summary of pneumonia and mortality measures. Of the 10,687 non-severe pneumonia cases, 29.52% were missing at least one parameter of interest. The overlap in classification criteria is detailed in the Venn diagram provided in Additional file 3.

For the outcome of interest — inpatient mortality — the prevalence rate was 2.36% (*N* = 252), with 0.82% (*N* = 93) of the non-severe pneumonia cases having missing outcome data and 538 cases missing pneumonia severity classification. Only 10,687 non-severe pneumonia cases were used in subsequent analysis, as illustrated in Fig. 1. The variables in Table 1 were included in an adjusted logistic regression model, with the results reported in Table 2.

**Table 2:** Predictors of inpatient mortality of non-severe pneumonia in children under 5 years

Predictors	Adjusted odds ratio (95% confidence interval)	*p* value
Age < 12 months (*Ref: ≥ 12 months*)	2.89 (2.17–3.85)	<0.001
Female (*Ref: Male*)	1.52 (1.17–1.96)	0.002
Respiratory rate ≥ 70 breaths/min (*Ref: < 70*)	2.49 (1.91–3.25)	<0.001
Temperature ≥ 39 °C (*Ref: < 39 °C*)	1.98 (1.38–2.84)	<0.001
Pallor (*Ref: No pallor*)		
Mild/moderate pallor	4.36 (2.88–6.58)	<0.001
Severe pallor	4.37 (2.13–8.96)	<0.001
Some dehydration (*Ref: No dehydration*)	1.06 (0.67–1.67)	0.819
WAZ (Ref: *Normal WAZ*)		
Low WAZ	2.08 (1.48–2.92)	<0.001
Very low WAZ	3.66 (2.59–5.18)	<0.001
Hospital in malaria endemic area	1.3 (1–1.69)	0.047
Non-severe malaria (*Ref: No malaria*)	0.81 (0.52–1.27)	0.36
Presence of comorbidity^a^	1.91 (1.4–2.6)	<0.001

*N* = 10,687. Outcome = inpatient mortality

*WAZ* weight-for-age Z-score

^a^Admission diagnosis of malaria, diarrhoea, dehydration and anaemia considered

Children less than 1 year old and those with respiratory rate ≥ 70 breaths/min each had three times the odds of mortality compared to their comparators. The presence of any level of pallor was associated with the largest odds of inpatient mortality, with children having even mild/moderate pallor experiencing at least four times the odds of inpatient mortality compared to children without pallor. Of the features selected for these analyses, only those associated with a diagnosis of malaria and dehydration were associated with no statistically significant effect on inpatient mortality.

### Results from machine learning approaches

Across the machine learning techniques used, *Age < 12 months*, *Respiratory rate ≥ 70 breaths/min, Presence of comorbidities, Female sex* and *Very low WAZ* consistently featured among the topmost five features explaining variability in inpatient mortality (see Table 3). It is noteworthy that the sizes of the odds ratio from Table 2 do not correspond with the order of feature importance in Table 3 below — a common assumption made when generating risk scores from logistic models. The reason is that the estimation of the contribution of each variable to the logistic model is determined by its corresponding *t* statistic absolute value. Detailed explanations of variable importance estimation for each of the models included in the analysis are reported elsewhere [26] and demonstrated in the analysis codes provided in Additional file 4.

**Table 3:** Variable importance ranking for the models used in the analysis of WHO-defined non-severe pneumonia

Model		Logistic	PLS-DA	Random forest	Elastic net	Linear SVM	Mean rank (SD)
Feature importance rank	Respiratory rate ≥ 70 breaths/min	3	1	1	2	3	2 (0.89)
	Age < 12 months * Respiratory rate ≥ 70	2	2	8	8	2	4.4 (2.94)
	Age < 12 months	1	3	7	11	1	4.6 (3.88)
	Very low WAZ	7	5	3	6	7	5.6 (1.5)
	Presence of comorbidity	5	4	6	10	5	6 (2.1)
	Female	6	7	2	9	6	6 (2.28)
	Mild/moderate pallor	8	9	4	3	8	6.4 (2.42)
	Temperature > 39 °C	4	13	10	-	4	7.75 (3.9)
	Severe pallor	13	8	5	1	13	8 (4.65)
	Malaria endemic	12	6	9	13	12	10.4 (2.58)
	Malaria endemic * Mild/moderate pallor	11	14	16	4	11	11.2 (4.07)
	Comorbidity * Very low WAZ	10	12	13	-	10	11.25 (1.3)
	Non-severe malaria	16	10	11	7	16	12 (3.52)
	Some dehydration	9	15	18	15	9	13.2 (3.6)
	Low WAZ	14	16	12	-	14	14 (1.41)
	Comorbidity * Low WAZ	17	17	15	5	17	14.2 (4.66)
	Malaria endemic * Non-severe malaria	18	11	14	12	18	14.6 (2.94)
	Malaria endemic * Severe pallor	15	18	17	14	15	15.8 (1.47)

Variables with (*) are interaction terms, i.e. variables used to test whether the effect of one independent variable differed depending on the level of the other independent variable

*SD* standard deviation

**Fig. 2:**
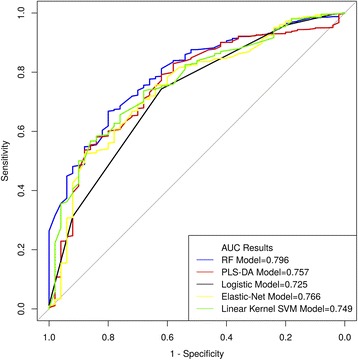
Receiver operating characteristic's area under curve (AUC) illustrating the model performance of different machine learning models. The best AUC is the one closest to 1

The performance as represented by AUC score was consistent across the selected models (Fig. 2), ranging from 0.725 to 0.796. These models were all found to be moderately discriminative for inpatient mortality risk. Using the full dataset with imputation in modelling, logistic and random forest models demonstrated much higher prediction accuracy (>0.9) than the other models but had low sensitivity (see Additional file 2: Table S4).

**Fig. 3:**
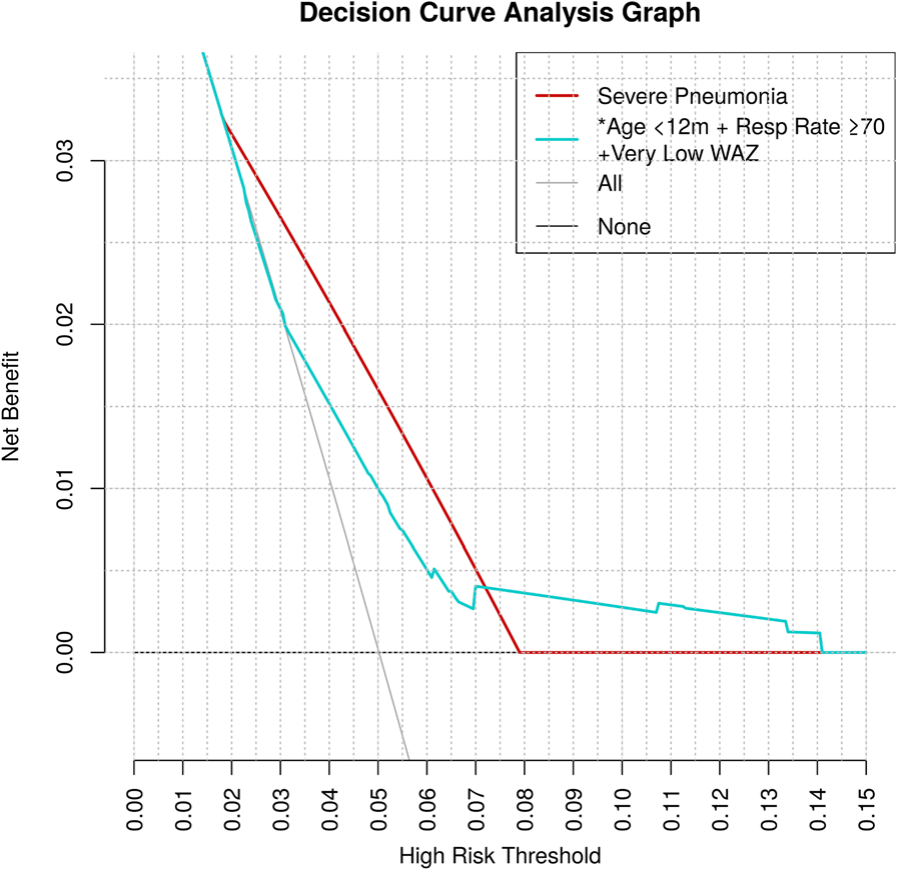
Decision curve analysis for the cohort of pneumonia paediatric patients. *Models are for WHO 2013 non-severe pneumonia definition except the one representing severe pneumonia indicator. The models that perform best are those to the extreme right of the figure

Overall, while all models had a higher AUC score than traditional logistic regression models (Fig. 3), from DeLong’s significance test, these differences were not statistically significant apart from the random forest model (see Additional file 5). However, the PLS-DA model demonstrated the highest sensitivity for inpatient mortality when using imputation and was least influenced when only complete cases were used. PLS-DA was therefore used for subsequent sensitivity analysis — this is consistent with the pragmatic perspective emphasising sensitivity over specificity across pneumonia to allow for optimisation of public health benefits [36]. For purposes of feature selection, the average feature rank across all models in Table 3 was used as a guide. From the table, *Respiratory rate ≥ 70 breaths/min, Age < 12 months* and *Very low WAZ* were the three features targeted for subsequent decision analysis due to their high average ranks.

### Decision-analytic results from evaluation of features selected

To evaluate the clinical impact of using the selected features in decisions to admit non-severe pneumonia patients in routine practice as part of screening algorithms, we performed decision curve analysis. The results of this analysis are presented in Fig. 3. The current decision to admit is based on whether the patient has a diagnosis of severe pneumonia — which is associated with a high mortality risk. We included an overlay of a model of severe pneumonia to help compare the net benefit of the decision to admit using the top three clinical and patient features identified compared to the current strategy (admit only severe pneumonia). The models that performed best are those to the extreme right of the figure.

Those to the right of the current admission criteria (severe pneumonia) are modelled on non-severe pneumonia patients based on WHO criteria. The threshold probability associated with the highest predicted inpatient mortality risk varied from 2 to 14%. At predicted probability thresholds between 2 and 7%, severe pneumonia model’s net benefit was greater than all other models and greater than strategies labelling all patients at high risk (grey line) or none at high risk (black line). For predicted probability thresholds between 7 and 14%, the net benefit of a model for non-severe pneumonia patients less than 12 months old with respiratory rate ≥ 70 breaths/min and very low WAZ was greater than that for all other models and greater than strategies labelling all patients at high risk (grey line) or none at high risk (black line). This is illustrated in Fig. 3. In summary, for a probability threshold of inpatient mortality between 7 and 14%, there is a net benefit for admitting non-severe cases of pneumonia involving children less than 12 months old with respiratory rate ≥ 70 breaths/min and with very low WAZ.

### Sensitivity analysis

**Fig. 4:**
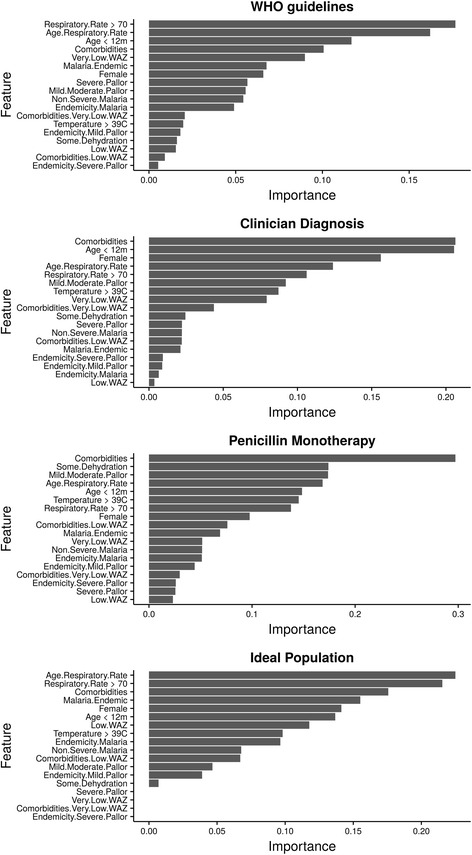
Variable (feature) importance ranking from PLS-DA model using different pneumonia severity classification criteria. More details are given in Additional file 2: Table S4

Given that the PLS-DA model had an AUC score higher than the logistic model (Fig. 2) and a sensitivity greater than all other models when considering its performance in both imputed and complete case analysis (see Additional file 2: Table S4), we used it for comparison of variable importance across the alternative criteria for pneumonia classification. This is guided by evidence from published studies showing AUC to be statistically consistent and a more discriminating measure than accuracy [37]. From Fig. 4, when WHO guidelines are used to determine the severity of pneumonia cases, the predictors that best explain the variance of inpatient mortality outcome among children with non-severe pneumonia are age, respiratory rate, comorbidities and very low WAZ.

This slightly differs from clinician admission diagnosis results where the presence of comorbidity is ranked higher than respiratory rate. In the non-severe pneumonia sub-population defined by prescription of penicillin monotherapy at admission criteria and also in the ideally diagnosed and managed sub-population, WAZ and age do not rank highly as risk factors. This might be indicative that in these sub-populations, very low WAZ and age explained very little variation in inpatient mortality.

From the results comparing whether risk factors were strongly linked to the pneumonia classification criteria, clinician classification criteria had the best performance in predicting inpatient mortality outcomes (see Additional file 2: Table S5). All pneumonia classification criteria, apart from that of the ideal population, had moderately acceptable performance with AUC > 0.7 (Additional file 2: Table S5). However, a comparison of the WHO classification criteria to each of the other pneumonia classification criteria revealed no statistically significant differences in AUC values (see Additional file 5). This is suggestive that differences in pneumonia classification criteria might not have a statistically significant impact in determining risk factors for this population.

## Discussion

### Summary findings

This study investigated the clinical and patient attributes that best discriminated the risk of inpatient mortality in children with non-severe pneumonia aged 2–59 months and the net clinical benefit of targeting these features using routine hospital data from a clinical information network running in 14 hospitals across Kenya. Several machine learning techniques were applied for discriminating these risks to test whether, given the available data, models using complex adaptive techniques performed better or provided robust ways to make decisions from model outputs, and also to see if there was consistency of findings across the alternative model choices.

Our analysis revealed age, respiratory rate and very low WAZ to be moderately discriminative of inpatient mortality in non-severe pneumonia cases across all models. Looking at the clinical consequences associated with features identified, the decision to admit these patients based on the characteristics identified was predicted to have a substantively higher net benefit on inpatient mortality outcomes for risk probability thresholds between 7 and 14% compared to using the existing WHO criteria for severe pneumonia only.

### Relation to other studies

Other studies conducted in similar epidemiological and geographical contexts have found comorbidities to be significant predictors of mortality in pneumonia patients, with odds being three times that of non-comorbid patients [38–42]. However, the study populations have consisted of both severe and non-severe pneumonia cases considered as a single population [38] or have omitted patients with any comorbidity [43].

Methodologically, our approach goes beyond the use of effect sizes for the determination of risk factors coupled with expert opinion [44]. In addition to traditional logistic regression models, we also applied robust machine learning techniques posited to improve modelling results by offering the ability to rank the importance of clinical signs and patient characteristics as risk factors for pneumonia mortality and also evaluate the consequence of using them in clinical decision making. These approaches have previously been used to predict risk of pneumonia and mortality, although not using a similar patient cohort or in a similar geographic context [45, 46].

While traditional statistical modelling techniques are simpler to implement and offer easier interpretation of results, machine learning techniques — which are adaptive to the datasets they are applied to and tend to perform better given their complex estimation procedures — are growing in popularity. However, these machine learning approaches produce results that are often regarded as difficult to interpret and operationalise due to their “black-box” nature [47]. It is becoming more difficult to decide which modelling approach to use, given the growing volume and veracity of clinical and epidemiological data. This is evident in our findings, where in predicting inpatient mortality, all models had a higher AUC and sensitivity compared to the traditional logistic regression model, with the exception of random forest’s sensitivity performance. Our approach was consistent with the pragmatic perspective adopted by WHO that emphasises sensitivity over specificity across pneumonia and other case definitions in this region in order to optimise public health benefits [36]. This ought to be — and was — factored into our choice of model.

### Implications of findings

The WHO guidelines recommend oxygen therapy for children with respiratory rates ≥ 70 breaths/min [48]. While this guidance implicitly suggests the need for admission care, this clinical sign is not listed among the classification criteria for severe pneumonia. In this study, respiratory rate ≥ 70 breaths/min was independently associated with increased inpatient mortality, and there was a net benefit of clinicians’ decision to admit non-severe pneumonia cases with tachypnoea at this threshold. The introduction of point-of-care diagnostic tools to objectively assess respiratory rates in routine care settings may mitigate the challenges of reliable measurement and the potential for misclassification of pneumonia severity on the basis of this sign [49].

The presence of pallor is associated with high risk of inpatient mortality in children with severe pneumonia [50] and, from our findings, in non-severe pneumonia also. This might be attributed to similarities in clinical presentation for malaria and severe pneumonia in children [51, 52], presenting challenges in discrimination of the two where confirmatory microbiological tools are unavailable. The findings of this study demonstrating increased risk of inpatient mortality in children presenting with any comorbidities are particularly relevant in SSA where comorbidities in non-severe pneumonia are high [53]. The adoption of treatment guidelines from controlled studies that fail to factor in common local comorbidities in children may be inappropriate in high mortality settings [54, 55].

Among demographic characteristics, female sex and age younger than 12 months were independently associated with increased odds of inpatient mortality. These risk factors were also highlighted in a recent review of 77 studies conducted in low- and middle-income countries [56]. While the risk associated with young age may be attributed to the limited capacity of the developing immune system to withstand severe infections [57], higher mortality among girls is less clear. Gender inequities in care seeking have been posited to play a role [58, 59]; however, the evidence to support this theory remains weak, warranting further study.

Despite having excluded children with documented admission diagnoses of severe acute malnutrition from the study population, analyses based on WAZ computed using available data on weight and age revealed increased mortality among children with low (–2 to –3SD) and very low (< –3SD) WAZ. These findings, consistent with the results of a large systematic review, challenge a recent technical update to the WHO guidelines for the management of severe acute malnutrition, which now recommends the use of weight-for-height Z-score and mid-upper arm circumference in place of WAZ for the diagnosis of severe malnutrition [60].

### Strengths, limitations and generalisability of the findings

The large sample size drawn from the hospitals across the country resulted in precise estimates that are representative of the population of children hospitalised with pneumonia in a majority of hospitals in Kenya. The 2-year data collection period further strengthened representativeness by eliminating seasonal bias — an important consideration in studies on acute respiratory infections in children [61].

Our data were limited by the clinical information network’s reliance on documented diagnoses in the absence of diagnostic tests. However, the approach adopted for data collection remains the only realistic data source that reflects how routine paediatric care is delivered at scale in Kenya. To mitigate this limitation, considerable efforts to improve data quality have been made, including follow-up in the laboratory to confirm whether there was evidence that investigations were performed and their results. Additionally, the data used reflect the routine practice of many clinicians in the Kenyan setting, thereby increasing the generalisability of our findings.

We restricted our analysis to children who were born during the period after the introduction of the pneumococcal vaccine to provide an understanding of the risk factors for pneumonia mortality in the current era. A consequence of this was the exclusion of older children who had not received the vaccine in the early period of the study and a relatively larger proportion of younger children with a higher risk of death.

Finally, the study sites were part of an ongoing implementation science project designed to improve quality of documentation practices and utilisation of data to improve care of children in district-level hospitals in Kenya. The population studied may therefore not be representative of children presenting to lower level health facilities or in the community where the influence of limited staff and resources and care-seeking behaviour may all influence clinical presentation and prognosis of pneumonia.

## Conclusions

Children aged 2–11 months with non-severe pneumonia, very low WAZ and respiratory rate ≥ 70 breaths/min have a risk of inpatient mortality higher than those with severe pneumonia for a risk threshold probability between 7 and 14%. These findings were obtained through use of cross-validated prediction modelling of clinical data from hospitals representing most regions of Kenya. The models had modest discriminative and calibration performance but performed much better than the traditional logistic regression. Our findings support the need for re-evaluation of the updated WHO guidelines for non-severe pneumonia, specifically among infants and in populations where comorbidities are common. This study also underlines the need for (re)calibration of pneumonia risk score models in their contexts of use.

## Additional files

Additional file 1: WHO paediatric pneumonia guidelines. *AVPU* represents altered consciousness state, with *A* Alert, *V* Verbal Response, *P* Pain Response and *U* Unresponsive. *RR* respiratory rate, *URTI* upper respiratory tract infection. (TIF 1021 kb)

Additional file 2: **Table S1.** Brief definitions and description of machine learning terms mentioned in the report. **Table S2.** Brief description of machine learning models used in analysis. **Table S3.** Description of Synthetic Minority Over-Sampling Technique (SMOTE) and how it has been applied in this paper. **Table S4.** Comparison of models’ performance between the imputed and complete case analysis. **Table S5.** PLS-DA model performance results for the different non-pneumonia classification criteria. (DOCX 28 kb)

Additional file 3: Non-severe pneumonia patients by classification criteria. (TIFF 3486 kb)

Additional file 4: R analysis code used in this study. (PDF 2966 kb)

Additional file 5: Test for statistical significance of the difference between two AUC-ROC curves. AUC values provided in Fig. 2, 95% confidence interval values are provided in Additional file 2: Table S4. (TIF 7372 kb)
