# Correction to: An exploration of mortality risk factors in non-severe pneumonia in children using clinical data from Kenya

**DOI:** 10.1186/s12916-017-0980-8

**Published:** 2017-12-05

**Authors:** Timothy Tuti, Ambrose Agweyu, Paul Mwaniki, Niels Peek, Mike English, on behalf of the Clinical Information Network Author Group

**Affiliations:** 1 KEMRI – Wellcome Trust Research Programme, Nairobi, Kenya; 2 Centre for Health Informatics, Division of Informatics, Imaging & Data Sciences, Faculty of Biology, Medicine and Health, The University of Manchester, Manchester Academic Health Science Centre, Manchester, UK; 3 NIHR Greater Manchester Primary Care Patient Safety Translational Research Centre, Manchester, UK; 4 Nuffield Department of Medicine, Oxford University, Oxford, UK

## Correction

The original article [1] contains an omission in the Acknowledgements sub-section of the Declarations.

The authors would like to acknowledge the work of the following members of the Clinical Information Network Author Group: David Githanga, Fred Were, Philip Ayieko, Grace Irimu, Sam Akech, Samuel Ng'arng'ar, Barnabas Kigen, Rachel Inginia, Nick Aduro, Grace Ochieng, Beatrice Mutai, Francis Kanyingi, Lydia Thuranira, Sam Otido, Magdalene Kuria, Peris Njiiri, Kigondu Rutha, Charles Nzioki, Martin Chabi, Supa Tonje, Joan Ondere, Caren Emadau, Cecelia Mutiso, Loice Mutai, Christine Manyasi, David Kimutai, Celia Muturi, Agnes Mithamo, Anne Kamunya, Alice Kariuki, Grace Wachira, Melab Musabi, Sande Charo, Naomi Muinga, Mercy Chepkirui, Wycliffe Nyachiro, Boniface Makone, Thomas Julius, George Mbevi, Morris Ogero, Susan Gachau, and James Wafula.
