# Supplementary material for: An exploration of mortality risk factors in non-severe pneumonia in children using clinical data from Kenya
Source: BMC Med. 2017 Nov 13;15:201. doi: 10.1186/s12916-017-0963-9 (PMC5682642; doi:10.1186/s12916-017-0963-9)
Supplement: Supplementary file 2 — Table S1. Brief definitions and description of machine learning terms mentioned in the report. Table S2. Brief description of machine learning models used in analysis. Table S3. Description of Synthetic Minority Over-Sampling Technique (SMOTE) and how it has been applied in this paper. Table S4. Comparison of models’ performance between the imputed and complete case analysis. Table S5. PLS-DA model performance results for the different non-pneumonia classification criteria. (DOCX 28 kb) [file 12916_2017_963_MOESM2_ESM.docx]

# **Supplementary Material for “****Exploration of mortality risk factors in non-severe pneumonia in children using clinical data from Kenya”**

## **Contents**

### Table S1………………………………………………………………………………2

### Table S2………………………………………………………………………………3

### Table S3………………………………………………………………………………5

### Table S4………………………………………………………………………………7

### Table S5………………………………………………………………………………8

| **Table S1: Machine Learning Definitions** | |
| --- | --- |
| Model Accuracy | The sum of true positive and true negative cases divided by the total number of cases. |
| Feature selection | Techniques within models that do not alter the original representation of the variables, but merely select a subset of them for use in model construction. The important objectives of feature selection are (a) to avoid overfitting and improve model performance, (b) to provide faster and more cost-effective models and (c) by reducing model complexity, make it easier gain a deeper insight into the underlying processes that generated the data and interpret them. |
| 10-fold cross-validation | Two-thirds of the analysis data was randomly partitioned into 10 equal size subsamples, with a single subsample retained as the validation data for testing the model, and the remaining nine subsamples used as training data. This randomised process was repeated five times with a different set of 9 training sets and one test set, with the results from these iterations averaged to produce a single estimation.  The number of folds was based on commonly used practice in machine learning in absence of gold standard |

| **Table S2: Brief Explanation of Machine Learning Models Used in the Analysis** | | |
| --- | --- | --- |
| **Model Type** | **Model Description** | **Critical Model Parameter Used for Analysis** |
| *Partial Least Square Discriminant Analysis (PLS-DA)* [23] | A classification modelling technique that identifies latent factors through principle component analysis. PLS-DA maximises the factors that correlate to response, then maps them directly to outcome by applying logistic regression. These technique is especially useful where measured predictors for any particular problem are highly correlated. | *Ncomp*=4 |
| Support Vector Machines (SVM) [25] | Classifiers dividing data instances of different categories with a linear boundary supported by a very clear gap (called maximum margin). They are optimised using different internal algorithms and therefore a parameter search is often recommended. SVM can also perform non-linear classifications by mapping their inputs into feature spaces of higher dimensions. The results however are difficult to interpret: it is a so-called "black box" classification method. In this study, we adopted the linear kernel SVM model. This would allow for the model to factor in the weight vector of the linear classifiers which are indicative of feature importance. | *Cost* =4 |
| Random Forests (RF) [24] | A well-recognised decision tree ensemble method, infers different decision trees via resampling and randomization, and produces an average prediction of all trees. Decision trees are non-linear models that use of a flow chart from to produce easily interpretable multiple decision pathways. They consist of nodes which represent input variables, and edges branching from the nodes dependent on possible values of those input variables. Each terminal node (leaf) represents the value of the target variable given the values of the input variables after following the path from the root to the leaf. A decision tree is usually grown by starting from the whole population, looking at the most discriminative variable to predict a desired outcome (which becomes a node), and splitting the data based on a cut-off value of this variable (inducing an edge). | *Mtry*=3  *Ntree*=900 |
| *Elastic Net* [26] | a hybrid regression approach that blends both penalization and regularisation of regression coefficients in prognostic models (48, 49). It combines the ability to shrink regression coefficients while automatically performing variable selection for correlated variables and removes any degeneracies while catering for unexpected behaviour caused by extreme variable correlations. This improves predictive performance and introduces parsimony. | *Lambda*=0.1,  *Alpha*=0.1178571 |
| *Note: The explanations given of the models used are not meant to be detailed - which we leave to the referenced works - but to offer introduction to techniques which may be less familiar* | | |

| **Table S3: Synthetic Minority Over-Sampling Technique (SMOTE)** | |
| --- | --- |
| Definition | Function that oversamples the rare event by using bootstrapping and k-nearest neighbour to synthetically create additional observations of that event. A rare event is usually attributed to any outcome response variable that occurs less than 15% of the time. |
| ***Parameters*** | |
| Data | A data frame of all predictors containing the original (unbalanced) data set. In our case, all predictors of the train dataset were used in the resampling technique. |
| Percent Over = 200% | Drives the decision of how many extra cases from the minority class are generated (known as over-sampling). In our model, this was set to 200% i.e. X2. |
| Percent Under = 200% | Drives the decision of how many extra cases from the majority classes are selected for each case generated from the minority class (known as under-sampling), this was set to 200% i.e. X0.5 |
| k = 5 | The number indicating the number of nearest neighbours that are used to generate the new examples of the minority class (Outcome=Death). In our analysis, 5 cases were used. |
| ***Mechanism*** | |
| How nearest neighbours are determined | The positive cases (minority class) is over-sampled by taking each positive case sample and introducing synthetic examples along the line segments joining any/all of the k(5) positive case nearest neighbours. Neighbours from the k(5) nearest neighbours are randomly chosen. |
| How information from nearest neighbours is combined with the information from original negative cases.  How much of the information is used | Since the amount of over-sampling needed is 200%, only two neighbours from the five nearest neighbours are chosen and one sample is generated in the direction of each.  Synthetic samples are generated in the following way: Take the difference between the feature vector (sample) under consideration and its nearest neighbour. Multiply this difference by a random number between 0 and 1, and add it to the feature vector under consideration. |
| ***Dealing with possible bias*** | |
| *Challenge:* Sample data from before model fitting | 1. During model tuning, the holdout samples generated during resampling may not reflect the class imbalance that future predictions would encounter. This is likely to lead to overly optimistic estimates of performance.  2. The subsampling process has a high probability of inducing more model uncertainty. Model results may differ under a different subsample and as a result, the resampling statistics are more likely to make the model appear more effective than it actually is. |
| *Our Solution:* Include subsampling inside the usual resampling procedure | This is advocated for in pre-process and feature selection steps. The subsampling happens within the 10-fold cross-validation. This is shown to have results that are more consistent with the original dataset. <https://topepo.github.io/caret/subsampling-for-class-imbalances.html> |

| **Table S4: Model performance in predicting mortality in non-severe pneumonia** | | | | | |
| --- | --- | --- | --- | --- | --- |
| **Classification** | **Logistic** | **PLS-DA** | **RF** | **Elastic Net** | **SVM** |
| ***Analysis results from imputed dataset*** | | | | | |
| Accuracy | 0. 9392 | 0.7013 | 0.9338 | 0.7536 | 0.735 |
| 95% CI | 0.9281 - 0.949 | 0.6836 - 0.7186 | 0.9237 - 0.943 | 0.7369-0.7699 | 0.7178-0.7516 |
| Sensitivity | 0.34 | **0.68** | 0.22 | 0.6 | 0.64 |
| Specificity | 0.9475 | 0.7017 | 0.9474 | 0.7568 | 0.7368 |
| AUC (95% CI) | 0.725(0.658-0.792) | 0.757(0.691-0.823) | 0.796(0.742-0.85) | 0.766(0.705-0.827) | 0.749(0.68-0.817) |
|  | | | | | |
| ***Analysis results from complete case dataset*** | | | | | |
| Accuracy | 0.7677 | 0.7694 | 0.874 | 0.8828 | 0.7843 |
| 95% CI | 0.7476 - 0.787 | 0.7493 - 0.7886 | 0.8578 - 0.8889 | 0.8671-0.8972 | 0.7646-0.803 |
| Sensitivity | 0.64 | 0.52 | 0.52 | 0.36 | 0.48 |
| Specificity | 0.7695 | 0.7729 | 0.8789 | 0.89 | 0.7885 |
| AUC (95% CI) | 0.746(0.665-0.828) | 0.711(0.615-0.808) | 0.788(0.708-0.868) | 0.705(0.611-0.798) | 0.689(0.574-0.803) |
| *Note: CI = Confidence Interval. Pneumonia severity classification based on WHO criterion.* | | | | | |

| **Table S5: PLS-DA performance by Pneumonia Classification Criterion** | | | | |
| --- | --- | --- | --- | --- |
| **Classification** | **WHO Guidelines** | **Clinician Diagnosis** | **Penicillin Monotherapy** | **Ideal Population** |
| Accuracy | 0.7013 | 0.7803 | 0.8189 | 0.829 |
| 95% CI | 0.6836 - 0.7186 | 0.7659 – 0.7942 | 0.796-0.8403 | 0.8021-0.8537 |
| Cases* (N) | 8,105 | 10,152 | 3,649 | 2,589 |
| Mortality rate | 1.91% | 2.36% | 0.74% | 0.73% |
| AUC (95% CI) | 0.757(0.691-0.823) | 0.774(0.726-0.821) | 0.723(0.576-0.87) | 0.643(0.412-0.874) |
| Sensitivity | **0.68** | 0.6049 | 0.3333 | 0.4 |
| Specificity | 0.7017 | 0.7846 | 0.8226 | 0.8317 |
| *Note: CI = Confidence Interval.* | | | | |
