# Supplementary material for: An exploration of mortality risk factors in non-severe pneumonia in children using clinical data from Kenya
Source: BMC Med. 2017 Nov 13;15:201. doi: 10.1186/s12916-017-0963-9 (PMC5682642; doi:10.1186/s12916-017-0963-9)
Supplement: Supplementary file 4 — R analysis code used in this study. (PDF 2966 kb) [file 12916_2017_963_MOESM4_ESM.pdf]

Additional file 4

R analysis code for the pneumonia risk factors and decision curve analaysis

```
list.of.packages=c("boot","gtools","reshape2","MASS","class","data.table","caret",
"ROCR","ggplot2","doParallel","knitr","DMwR","rmda","klaR","kernlab","glmnet", "mi
ce")
new.packages = list.of.packages[!(list.of.packages %in% installed.packages()[,"Pac
kage"])]
if(length(new.packages)) install.packages(new.packages,dependencies=T)

suppressMessages({
require(boot)
library(gtools)
library(reshape2)
require(MASS)
library(class)
library(data.table)
library(caret)
library(ROCR)
library(ggplot2)
library(doParallel)
library(caTools)
library(pls)
library(kernlab)
library(randomForest)
library(knitr)
library(glmnet)
library(pROC)
library(cowplot)
library(DMwR)
library(rmda)
library(mice)
library(VennDiagram)
})

cl=makePSOCKcluster(detectCores()-1)
registerDoParallel(cl)
```

1. Descriptive Statistics

```
load(file="pneumonia.descriptive.rda")
pneumonia.rp=pneumonia.rp[!is.na(outcome) & !(pen.pres=="No" & pneum.severity=="Ve
ry Severe" & (clinician.diagnosis=="Pneumonia(No classification)[ICD10: J18.9]" |
clinician.diagnosis=="Pneumonia(Very severe)[ICD10: J18.9C]")),copy(.SD)]

pneumonia.rp[,cormobidity:=ifelse(cormobidity > 0, 1, 0)]
pneumonia.rp[,cormobidity:=factor(cormobidity,levels = 0:1,labels = c("No","Yes"))
]
pneumonia.rp[,c("los", "lill", "oxygen.sat", "pulse.rate", "pcv.doses"):=NULL]

i=1
generate.stats <- function(column.data,col.name=NA,weights=NA){
  idx=get("i",.GlobalEnv)
  col.name.i = col.name[idx]
  idx=idx+1
  assign("i",idx,.GlobalEnv)
  if(class(column.data) %in% c("integer","numeric")){
    mean.stat <- round(mean(x=column.data,na.rm=T),2)
    range.stat <- paste0(" (",range(column.data,na.rm=T)[1]," - ",range(column.dat
a,na.rm=T)[2],")")
    ret=data.frame(Indicator=c(col.name.i,"Missing"),
                   Levels=c("",""),
                   Value=c(paste0(mean.stat,range.stat),
                           paste0(sum(is.na(column.data))," (",paste0(round((sum(i
s.na(column.data))/length(column.data))*100,2),"%",")"))))
    return(ret)
  }

  if(class(column.data) %in% c("factor","character")){
    lvl.stats <- table(column.data,useNA="ifany")
    lvl.stats.prop <- round(prop.table(lvl.stats),4)*100
    ret.str <- paste0(lvl.stats,"(",lvl.stats.prop,"%)")
    ret=data.frame(Indicator=c(col.name.i,
                               rep("",length(ret.str)-1)),
                   Levels=names(lvl.stats),
                   Value=ret.str)
    return(ret)
  }
}
res.stat=lapply(pneumonia.rp,generate.stats,col.name=names(pneumonia.rp))
i=1
res = do.call(rbind,res.stat)
row.names(res)=NULL
res$Levels=as.character(res$Levels)
res$Levels[is.na(res$Levels)]="Missing"
names(res)[names(res)=="Value"]="N (%)"
res$Indicator=as.character(res$Indicator)
res$Indicator=c("Pallor", "", "", "", "Outcome", "", "Respiratory Rate ≥ 70 breath
es/minute", "","Weight for Age Z-Score (WAZ)", "", "", "", "Pneumonia severity (WH
O guidelines)", "", "", "", "Temperature ≥ 39 °C","", "", "Female", "", "", "Dehyd
ration", "", "", "", "Malaria", "", "", "Hospital located in malaria endemic area"
, "", "Acute Malnutrition", "", "Clinician pneumonia diagnosis","", "", "", "Penic
illin Monotherapy prescribed", "", "Age < 12 months", "", "Presence of comorbidity
","")
```

```
res$Levels[30]="Yes"
res$Levels[31]="No"

kable(res)
```

Indicator	Levels	N (%)
Pallor	Mild/Moderate	374(3.5%)
	None	7613(71.24%)
	Severe	98(0.92%)
	Missing	2602(24.35%)
Outcome	Alive	10435(97.64%)
	Dead	252(2.36%)
Respiratory Rate ≥ 70 breathes/minute	No	6622(61.96%)
	Yes	4065(38.04%)
Weight for Age Z-Score (WAZ)	Moderate	1202(11.25%)
	None/Mild	8311(77.77%)
	Severe	719(6.73%)
	Missing	455(4.26%)
Pneumonia severity (WHO guidelines)	Non-severe	1960(18.34%)
	Severe	6145(57.5%)
	Very Severe	2135(19.98%)
	Missing	447(4.18%)
Temperature ≥ 39 °C	No	6577(61.54%)
	Yes	1257(11.76%)
	Missing	2853(26.7%)
Female	No	5856(54.8%)
	Yes	4736(44.32%)
	Missing	95(0.89%)
Dehydration	No dehydration	10026(93.81%)
	Severe dehydration/shock	0(0%)
	Some dehydration	622(5.82%)
	Missing	39(0.36%)
Malaria	No malaria	9611(89.93%)
	Non-severe malaria	1076(10.07%)

Indicator	Levels	N (%)
	Severe malaria	0(0%)
Hospital located in malaria endemic area	Yes	4447(41.61%)
	No	6240(58.39%)
Acute Malnutrition	Moderate	115(1.08%)
	None/at risk	10572(98.92%)
Clinician pneumonia diagnosis	Pneumonia(No classification)[ICD10: J18.9]	260(2.43%)
	Pneumonia(Non Severe)[ICD10: J18.9A]	2395(22.41%)
	Pneumonia(severe)[ICD10: J18.9B]	7757(72.58%)
	Pneumonia(Very severe)[ICD10: J18.9C]	275(2.57%)
Penicillin Monotherapy prescribed	No	7038(65.86%)
	Yes	3649(34.14%)
Age < 12 months	No	5719(53.51%)
	Yes	4968(46.49%)
Presence of comorbidity	No	7330(68.59%)
	Yes	3357(31.41%)

2. Analysis with imputed data sets

```
load(file="pneumonia.rda")

pneumonia=pneumonia[,copy(.SD),.SDcols=c("age.lt.12m","female","tachypnoea","high.
fever","pneum.severity.Non.severe","pneum.severity.Severe","pneum.severity.Very.Se
vere","pallor.Mild.Moderate","pallor.Severe","dehydration.Some.dehydration","waz.M
oderate","waz.Severe","mal.end","malaria.Non.severe.malaria","cormobidity","mortal
ity","ns.pneum","clinician.diagnosis")]



pneumonia[,cormobidity:=ifelse(cormobidity > 0, 1, 0)]
pneumonia[,`None Severe`:=0]
pneumonia[pneum.severity.Non.severe==1 | pneum.severity.Severe==1,`None Severe`:=1
]
pneumonia[,pneum.severity.Non.severe:=NULL]
pneumonia[,pneum.severity.Severe:=NULL]
pneumonia[!is.na(clinician.diagnosis),clinician.diagnosis:=ifelse(clinician.diagno
sis==1,NA_real_,
                                                                ifelse(clinician
.diagnosis==2,1,
                                                                      ifelse(cl
inician.diagnosis==3,1,0)))]

pneumonia = pneumonia[,lapply(.SD,as.character)]
pneumonia = pneumonia[,lapply(.SD,as.numeric)]

setnames(pneumonia,
         c("age.lt.12m","female","high.fever","pneum.severity.Very.Severe","tachyp
noea","waz.Moderate","waz.Severe","dehydration.Some.dehydration","mortality","pall
or.Mild.Moderate","pallor.Severe","ns.pneum","clinician.diagnosis","None Severe","
mal.end","malaria.Non.severe.malaria" , "cormobidity"),
         c("Age.lt.12m","Female","Temperature.gt.39C","Severe.Pneumonia","Respirat
ory.Rate.gt.70","Low.WAZ","Very.Low.WAZ","Some.Dehydration","Mortality","Mild.Mode
rate.Pallor","Severe.Pallor","Severity.Treatment","Clinician.Diagnosis","None.Seve
re","Malaria.Endemic","Non.Severe.Malaria","Comorbidities"))

pneumonia.copy = pneumonia[,copy(.SD)]
```

2.1 Traditional Logistic regression output (with imputation)

```
pneumonia.log= pneumonia[!is.na(Mortality)& (None.Severe==1 | Clinician.Diagnosis=
=1 | Severity.Treatment==1),copy(.SD)]
pneumonia.log[,c("Severity.Treatment","Clinician.Diagnosis","None.Severe","Severe.
Pneumonia"):=NULL]
Comorbidities= pneumonia.log$Comorbidities
pneumonia.log=pneumonia.log[,lapply(.SD,factor)]
pneumonia.log$Comorbidities=Comorbidities
pneumonia.log.imp = mice(pneumonia.log,m=10,printFlag=F, maxit = 40, seed=197)
model.log = glm.mids(Mortality ~ ., data=pneumonia.log.imp, family = binomial(link
="logit"))
model.log = pool(model.log)

kable(summary(model.log)[,c(1:2,5)], digits=4)
```

	est	se	Pr(>|t|)
(Intercept)	-5.9110	0.2001	0.0000
Age.lt.12m2	1.0609	0.1462	0.0000
Female2	0.4163	0.1318	0.0016
Respiratory.Rate.gt.702	0.9120	0.1358	0.0000
Temperature.gt.39C2	0.6845	0.1813	0.0003
Mild.Moderate.Pallor2	1.4714	0.2106	0.0000
Severe.Pallor2	1.4750	0.3665	0.0001
Some.Dehydration2	0.0538	0.2349	0.8189
Low.WAZ2	0.7310	0.1736	0.0000
Very.Low.WAZ2	1.2987	0.1766	0.0000
Malaria.Endemic2	0.2628	0.1325	0.0473
Non.Severe.Malaria2	-0.2078	0.2271	0.3602
Comorbidities	0.6453	0.1576	0.0000

2.2 Machine Learning techniques that implement implicit feature selection

```
#Include interaction terms
suppressMessages({

  pneumonia[,Age.Respiratory.Rate:=ifelse(!is.na(Age.lt.12m) & !is.na(Respiratory.
Rate.gt.70),0,NA_integer_)]
  pneumonia[Age.Respiratory.Rate==0 & Age.lt.12m==1 & Respiratory.Rate.gt.70==1,Ag
e.Respiratory.Rate:=1]

  pneumonia[,Comorbidities.Low.WAZ:=ifelse(!is.na(Comorbidities) & !is.na(Low.WAZ)
,0,NA_integer_)]
  pneumonia[Comorbidities.Low.WAZ==0 & Comorbidities==1 & Low.WAZ==1,Comorbidities
.Low.WAZ:=1]

  pneumonia[,Comorbidities.Very.Low.WAZ:=ifelse(!is.na(Comorbidities) & !is.na(Ver
y.Low.WAZ),0,NA_integer_)]
  pneumonia[Comorbidities.Very.Low.WAZ==0 & Comorbidities==1 & Very.Low.WAZ==1,Com
orbidities.Very.Low.WAZ:=1]

  pneumonia[,Endemicity.Malaria:=ifelse(!is.na(Malaria.Endemic) & !is.na(Non.Sever
e.Malaria),0,NA_integer_)]
  pneumonia[Endemicity.Malaria==0 & Malaria.Endemic==1 & Non.Severe.Malaria==1,End
emicity.Malaria:=1]

  pneumonia[,Endemicity.Mild.Pallor:=ifelse(!is.na(Malaria.Endemic) & !is.na(Mild.
Moderate.Pallor),0,NA_integer_)]
  pneumonia[Endemicity.Mild.Pallor==0 & Malaria.Endemic==1 & Mild.Moderate.Pallor=
=1,Endemicity.Mild.Pallor:=1]

  pneumonia[,Endemicity.Severe.Pallor:=ifelse(!is.na(Malaria.Endemic) & !is.na(Sev
ere.Pallor),0,NA_integer_)]
  pneumonia[Endemicity.Severe.Pallor==0 & Malaria.Endemic==1 & Severe.Pallor==1,En
demicity.Severe.Pallor:=1]

  setcolorder(pneumonia,c("Age.lt.12m","Female","Respiratory.Rate.gt.70","Temperat
ure.gt.39C","Severe.Pneumonia","Mild.Moderate.Pallor","Severe.Pallor","Some.Dehydr
ation","Low.WAZ","Very.Low.WAZ","Malaria.Endemic","Non.Severe.Malaria","Comorbidit
ies","Severity.Treatment","Clinician.Diagnosis","None.Severe","Age.Respiratory.Rat
e","Comorbidities.Very.Low.WAZ","Comorbidities.Low.WAZ","Endemicity.Malaria","Ende
micity.Mild.Pallor","Endemicity.Severe.Pallor","Mortality"))
})

diagnosis=pneumonia[!is.na(Mortality) & (None.Severe==1 | Clinician.Diagnosis==1 |
Severity.Treatment==1),copy(.SD),.SDcols=c("None.Severe","Clinician.Diagnosis","Se
verity.Treatment")]
setnames(diagnosis,names(diagnosis),c("WHO.Guidelines","Clinician.Diagnosis","Peni
cillin.Monotherapy"))
diagnosis=diagnosis[,lapply(.SD,as.logical)]
venn.data=as.data.frame(matrix(c(
      0 ,0 ,0,
      1 ,0 ,0,
      0 ,1 ,0,
      0 ,0 ,1,
      1 ,1 ,0,
      1, 0, 1,
```

```
      0, 1, 1,
      1, 1, 1
    )
    ,nc = 3
    ,byrow = TRUE))
names(venn.data)=c("WHO.Guidelines","Clinician.Diagnosis","Penicillin.Monotherapy"
)
venn.data$Counts=0
venn.data$Counts[1]=diagnosis[!WHO.Guidelines & (!Clinician.Diagnosis | is.na(Clin
ician.Diagnosis)) & !Penicillin.Monotherapy,nrow(.SD)]
venn.data$Counts[2]=diagnosis[WHO.Guidelines & (!Clinician.Diagnosis | is.na(Clini
cian.Diagnosis)) & !Penicillin.Monotherapy,nrow(.SD)]
venn.data$Counts[3]=diagnosis[!is.na(Clinician.Diagnosis) & !WHO.Guidelines & Clin
ician.Diagnosis & !Penicillin.Monotherapy,nrow(.SD)]
venn.data$Counts[4]=diagnosis[!WHO.Guidelines & (!Clinician.Diagnosis | is.na(Clin
ician.Diagnosis)) & Penicillin.Monotherapy,nrow(.SD)]
venn.data$Counts[5]=diagnosis[WHO.Guidelines & Clinician.Diagnosis & !Penicillin.M
onotherapy,nrow(.SD)]
venn.data$Counts[6]=diagnosis[WHO.Guidelines & (!Clinician.Diagnosis | is.na(Clini
cian.Diagnosis)) & Penicillin.Monotherapy,nrow(.SD)]
venn.data$Counts[7]=diagnosis[!is.na(Clinician.Diagnosis) & !WHO.Guidelines & Clin
ician.Diagnosis & Penicillin.Monotherapy,nrow(.SD)]
venn.data$Counts[8]=diagnosis[WHO.Guidelines & Clinician.Diagnosis & Penicillin.Mo
notherapy,nrow(.SD)]

venn.data$Counts=paste0(venn.data$Counts,"(",round(venn.data$Counts/11225,4)*100,"
%)")

kable(venn.data)
```

WHO.Guidelines	Clinician.Diagnosis	Penicillin.Monotherapy	Counts
0	0	0	0(0%)
1	0	0	322(2.87%)
0	1	0	1592(14.18%)
0	0	1	143(1.27%)
1	1	0	5124(45.65%)
1	0	1	70(0.62%)
0	1	1	847(7.55%)
1	1	1	2589(23.06%)

Venn diagram of the diagnosis

```
invisible({
  draw.triple.venn(area1 = 8105, area2 = 10152, area3 = 3649, n12 = 7713, n23 = 34
36, n13 = 2659,
    n123 = 2589, category = c("WHO Guidelines","Clinician Diagnosis","Penicillin M
onotherapy"), lty = "blank",
    fill = c("skyblue", "pink1", "mediumorchid"),cat.fontfamily ="corbel",print.mo
de=c("raw","percent"),sigdigs=3,cat.dist = 0.005)
})
```

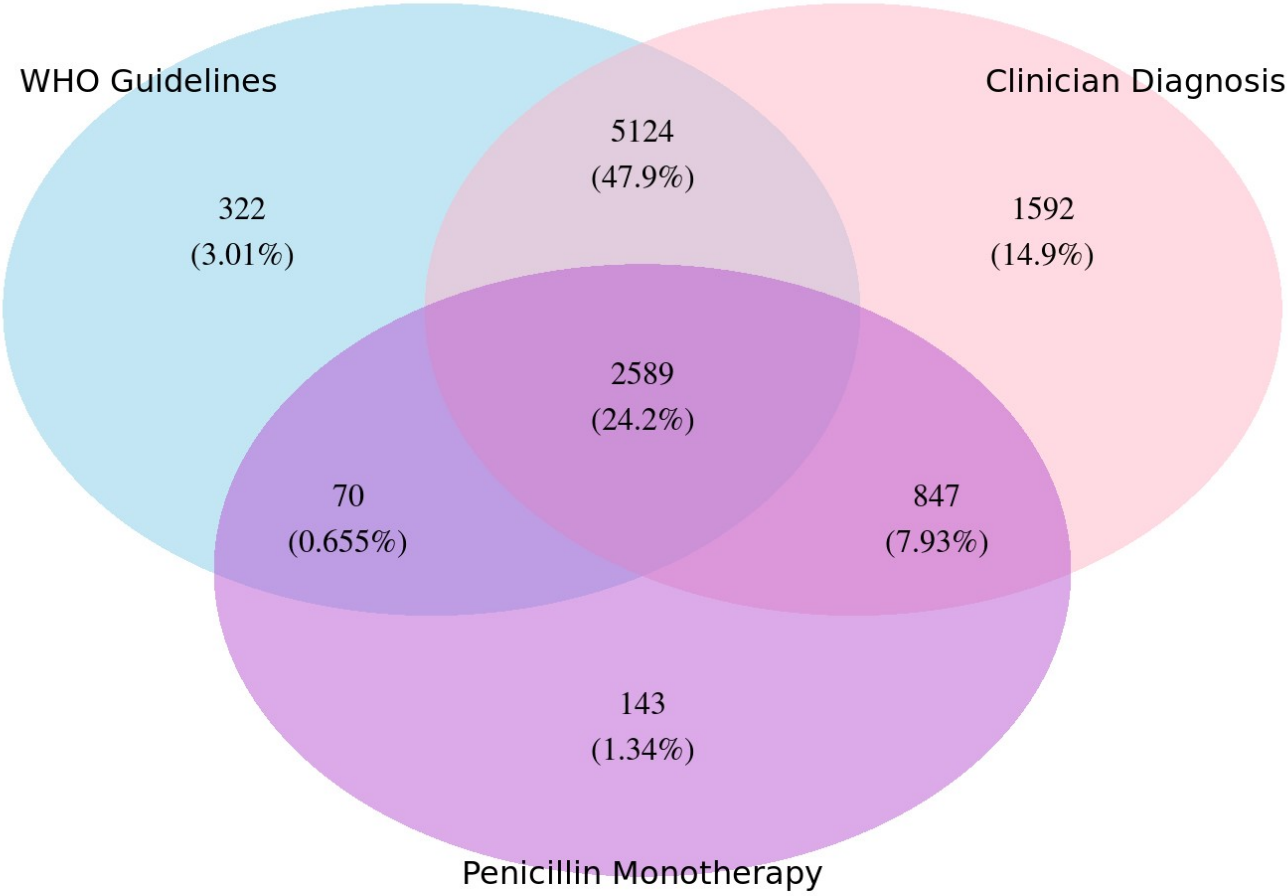


```
#Based on WHO guidelines
pneumonia.ns=pneumonia[None.Severe==1,copy(.SD)]
pneumonia.ns[,None.Severe:=NULL]
pneumonia.ns[,Clinician.Diagnosis:=NULL]
pneumonia.ns[,Severity.Treatment:=NULL]
pneumonia.ns[,Severe.Pneumonia:=NULL]

dataset=pneumonia.ns[!is.na(Mortality),copy(.SD)]

dataset$Mortality=factor(dataset$Mortality,levels=c(0,1),labels=c("Alive","Dead"))

dataset.tr=copy(dataset)
dataset=as.data.frame(dataset)
col.len=length(dataset)

paste0(round(prop.table(table(dataset$Mortality))*100,2)," %")
```

```
## [1] "98.09 %" "1.91 %"
```

```
set.seed(197)
train.ids=sample(seq(nrow(dataset)),round(nrow(dataset)*0.67),replace=F)

dataset.x=dataset[,1:(col.len-1)]
dataset.y=dataset[,col.len]

dataset.x.tr=dataset.x[train.ids,]
dataset.y.tr=dataset.y[train.ids]


dataset.x.ts=dataset.x[-train.ids,]
dataset.y.ts=dataset.y[-train.ids]

tune.len=length(dataset.x.tr)


##For complete cases
dataset.comp=copy(dataset.tr)
dataset.comp=dataset.comp[complete.cases(dataset.comp),copy(.SD)]
dataset.comp=as.data.frame(dataset.comp)

set.seed(197)
train.ids.comp=sample(seq(nrow(dataset.comp)),round(nrow(dataset.comp)*0.67),repla
ce=F)

dataset.x.comp=dataset.comp[,1:(col.len-1)]
dataset.y.comp=dataset.comp[,col.len]

dataset.x.tr.comp=dataset.x.comp[train.ids.comp,]
dataset.y.tr.comp=dataset.y.comp[train.ids.comp]

dataset.x.ts.comp=dataset.x.comp[-train.ids.comp,]
dataset.y.ts.comp=dataset.y.comp[-train.ids.comp]

tune.len=length(dataset.x.tr.comp)

##End data preparation for complete cases analysis
ctrl=trainControl(method="repeatedcv"
                  ,repeats=5
                  ,number=10
                  ,classProbs=T
                  ,allowParallel=T
                  ,search="random"
                  ,sampling = "smote"
                  ,returnResamp = "all"
                  ,summaryFunction=twoClassSummary)

ctrl.rf=trainControl(method="repeatedcv"
                     ,repeats=5
                     ,number=10
                     ,classProbs=T
                     ,search="grid"
                     ,allowParallel=T
                     ,sampling = "smote"
```

```
                      ,returnResamp = "all"
                      ,summaryFunction=twoClassSummary)
```

Logistic Regression Model with implicit feature selection(with imputed data)

```
set.seed(197)
train.model.log=train(y=dataset.y.tr
                      ,x=dataset.x.tr
                      ,method="LogitBoost"
                      ,trControl=ctrl
                      ,tuneGrid=expand.grid(nIter=seq(5,200,15))
                      ,importance=T
                      ,metric="ROC"
)
plot(train.model.log)
```

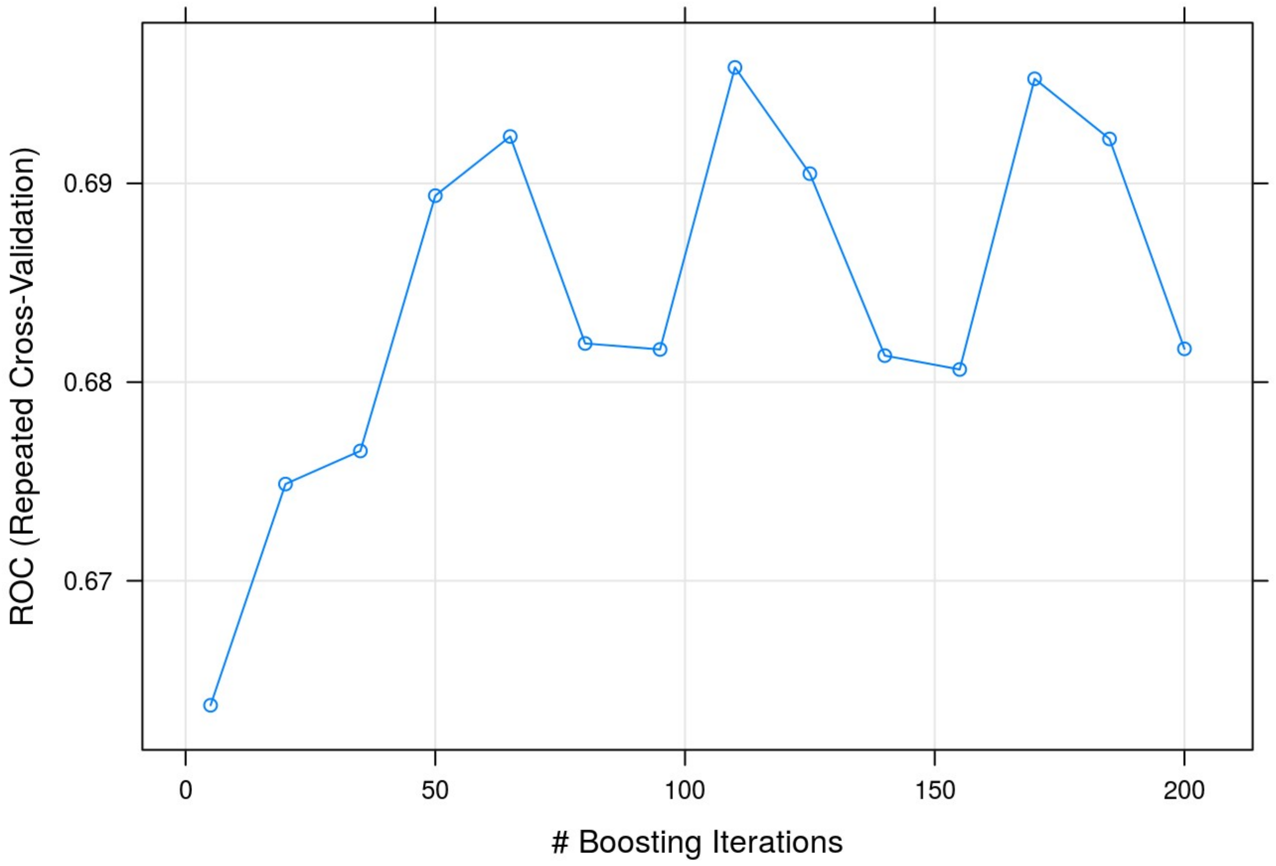


```
train.model.log$bestTune
```

```
##   nIter
## 8   110
```

Confusion matrix for logistic regression model (with imputed data)

```
pred.cm.log=predict(train.model.log,dataset.x.ts)
cm.log=confusionMatrix(pred.cm.log,dataset.y.ts,positive="Dead")
cm.log
```

```
## Confusion Matrix and Statistics
##
##           Reference
## Prediction Alive Dead
##      Alive  1951   19
##      Dead    108   10
##
##                Accuracy : 0.9392
##                  95% CI : (0.9281, 0.949)
##     No Information Rate : 0.9861
##     P-Value [Acc > NIR] : 1
##
##                   Kappa : 0.1164
##  Mcnemar's Test P-Value : 5.776e-15
##
##             Sensitivity : 0.344828
##             Specificity : 0.947547
##          Pos Pred Value : 0.084746
##          Neg Pred Value : 0.990355
##              Prevalence : 0.013889
##          Detection Rate : 0.004789
##    Detection Prevalence : 0.056513
##       Balanced Accuracy : 0.646187
##
##        'Positive' Class : Dead
##
```

PLS Model (with imputed data)

```
set.seed(197)
train.model.pls=train(x=dataset.x.tr
                      ,y=dataset.y.tr
                      ,method="pls"
                      ,trControl=ctrl
                      ,tuneLength = tune.len
                      ,importance=T
                      ,probMethod="Bayes"
                      ,metric="ROC"
                      ,preProc=c(method="bagImpute")
)
plot(train.model.pls)
```

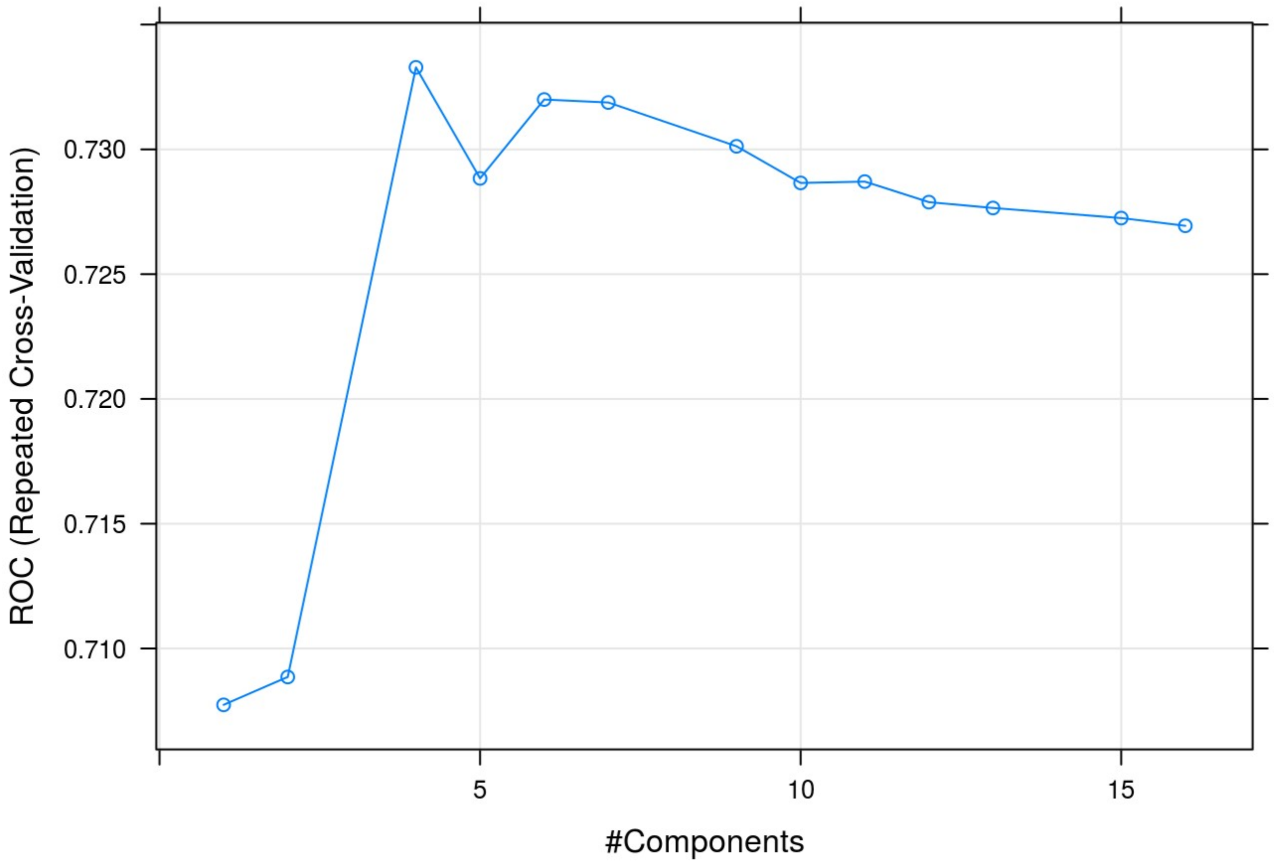


```
train.model.pls$bestTune
```

```
##   ncomp
## 3     4
```

Confusion matrix for PLS-DA model (with imputed data)

```
pred.cm.pls=predict(train.model.pls,dataset.x.ts)
cm.pls=confusionMatrix(pred.cm.pls,dataset.y.ts,positive="Dead")
cm.pls
```

```
## Confusion Matrix and Statistics
##
##           Reference
## Prediction Alive Dead
##      Alive  1842   16
##      Dead    783   34
##
##                Accuracy : 0.7013
##                  95% CI : (0.6836, 0.7186)
##     No Information Rate : 0.9813
##     P-Value [Acc > NIR] : 1
##
##                   Kappa : 0.0448
##  Mcnemar's Test P-Value : <2e-16
##
##             Sensitivity : 0.68000
##             Specificity : 0.70171
##          Pos Pred Value : 0.04162
##          Neg Pred Value : 0.99139
##              Prevalence : 0.01869
##          Detection Rate : 0.01271
##    Detection Prevalence : 0.30542
##       Balanced Accuracy : 0.69086
##
##        'Positive' Class : Dead
##
```

Support Vector Machine Model (with imputed data)

```
set.seed(197)
train.model.svm=train(x=dataset.x.tr
                      ,y=dataset.y.tr
                      ,method="svmLinear"
                      ,trControl=ctrl
                      ,tuneGrid=expand.grid(C=c(.0625,.125,.25,.5,1,2,4,8))
                      ,metric="ROC"
                      ,preProcess=c(method="bagImpute")
                      ,importance=T
                      ,probability=T
)
plot(train.model.svm)
```

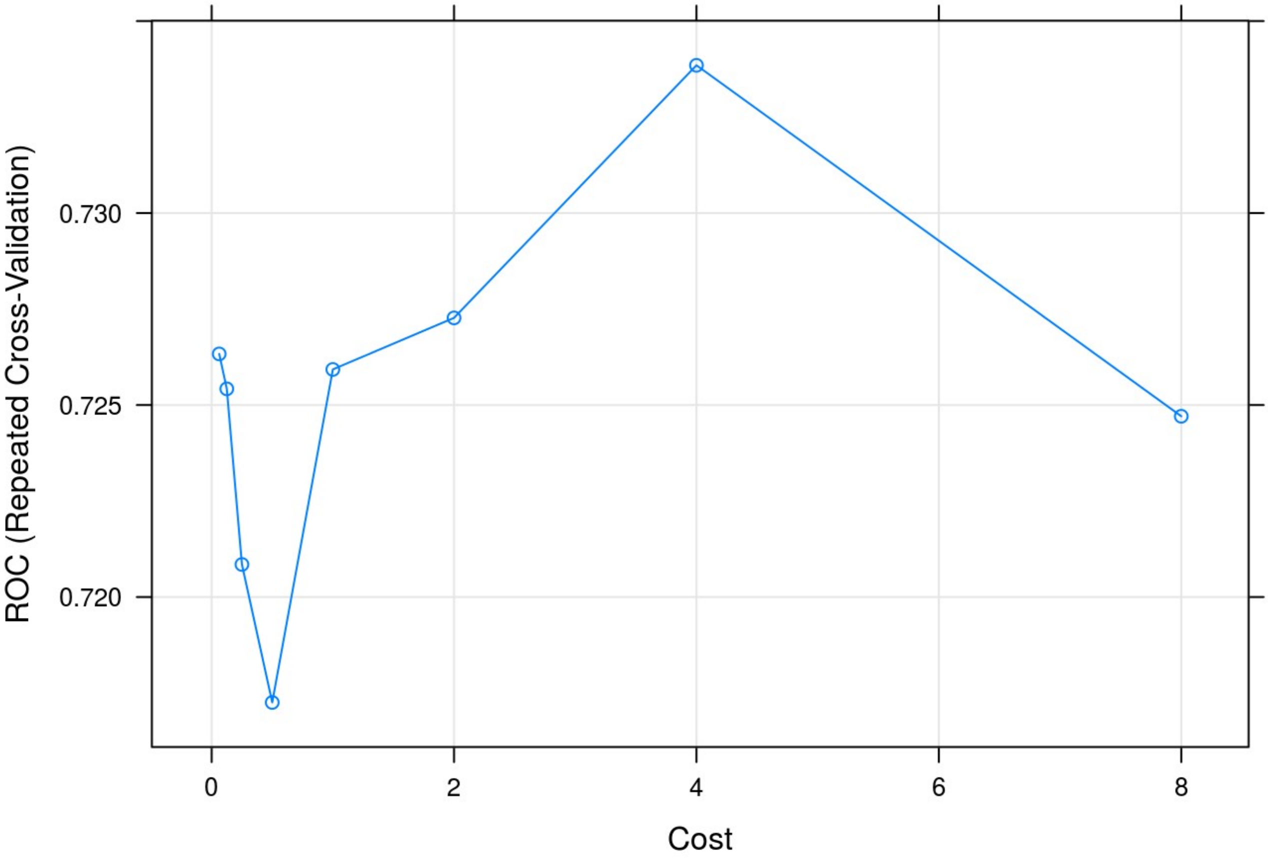


```
train.model.svm$bestTune
```

```
##   C
## 7 4
```

Confusion matrix for SVM model (with imputed data)

```
pred.cm.svm=predict(train.model.svm,dataset.x.ts)
cm.svm=confusionMatrix(pred.cm.svm,dataset.y.ts,positive="Dead")
cm.svm
```

```
## Confusion Matrix and Statistics
##
##           Reference
## Prediction Alive Dead
##      Alive  1934   18
##      Dead    691   32
##
##                Accuracy : 0.735
##                  95% CI : (0.7178, 0.7516)
##     No Information Rate : 0.9813
##     P-Value [Acc > NIR] : 1
##
##                   Kappa : 0.0496
##  Mcnemar's Test P-Value : <2e-16
##
##             Sensitivity : 0.64000
##             Specificity : 0.73676
##          Pos Pred Value : 0.04426
##          Neg Pred Value : 0.99078
##              Prevalence : 0.01869
##          Detection Rate : 0.01196
##    Detection Prevalence : 0.27028
##       Balanced Accuracy : 0.68838
##
##        'Positive' Class : Dead
##
```

Random Forest Model (with imputed data)

```
set.seed(197)
train.model.rf=train(x=dataset.x.tr
                     ,y=dataset.y.tr
                     ,method="rf"
                     ,trControl=ctrl.rf
                     ,tuneGrid=expand.grid(.mtry=c(1:tune.len))
                     ,importance=T
                     ,ntree=900
                     ,proximity=T
                     ,metric="ROC"
                     ,preProcess=c(method="bagImpute")
)
plot(train.model.rf)
```

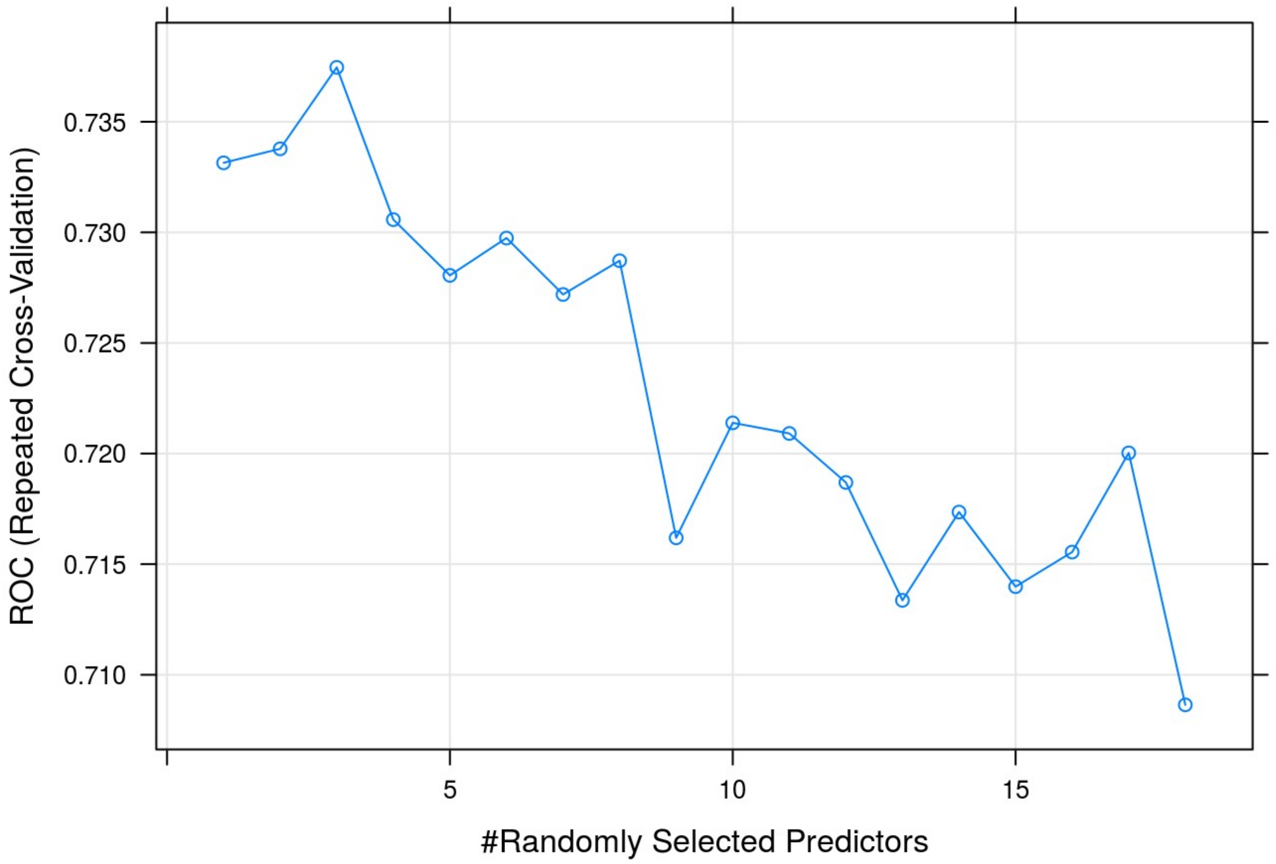


```
train.model.rf$bestTune
```

```
##   mtry
## 3    3
```

Confusion matrix for Random Forest model (with imputed data)

```
pred.cm.rf=predict(train.model.rf,dataset.x.ts)
cm.rf=confusionMatrix(pred.cm.rf,dataset.y.ts,positive="Dead")
cm.rf
```

```
## Confusion Matrix and Statistics
##
##           Reference
## Prediction Alive Dead
##      Alive  2487   39
##      Dead    138   11
##
##                Accuracy : 0.9338
##                  95% CI : (0.9237, 0.943)
##     No Information Rate : 0.9813
##     P-Value [Acc > NIR] : 1
##
##                   Kappa : 0.0849
##  Mcnemar's Test P-Value : 1.757e-13
##
##             Sensitivity : 0.220000
##             Specificity : 0.947429
##          Pos Pred Value : 0.073826
##          Neg Pred Value : 0.984561
##              Prevalence : 0.018692
##          Detection Rate : 0.004112
##    Detection Prevalence : 0.055701
##       Balanced Accuracy : 0.583714
##
##        'Positive' Class : Dead
##
```

Elastic Net Model (with imputed data)

```
set.seed(197)
train.model.net=train(x=dataset.x.tr
                      ,y=dataset.y.tr
                      ,method="glmnet"
                      ,trControl=ctrl
                      ,tuneGrid=expand.grid(.alpha = seq(.05, 1, length = 15),
                                           .lambda = c((1:5)/10))
                      ,metric="ROC"
                      ,family = "binomial"
                      ,preProcess=c(method="bagImpute")
)
plot(train.model.net)
```

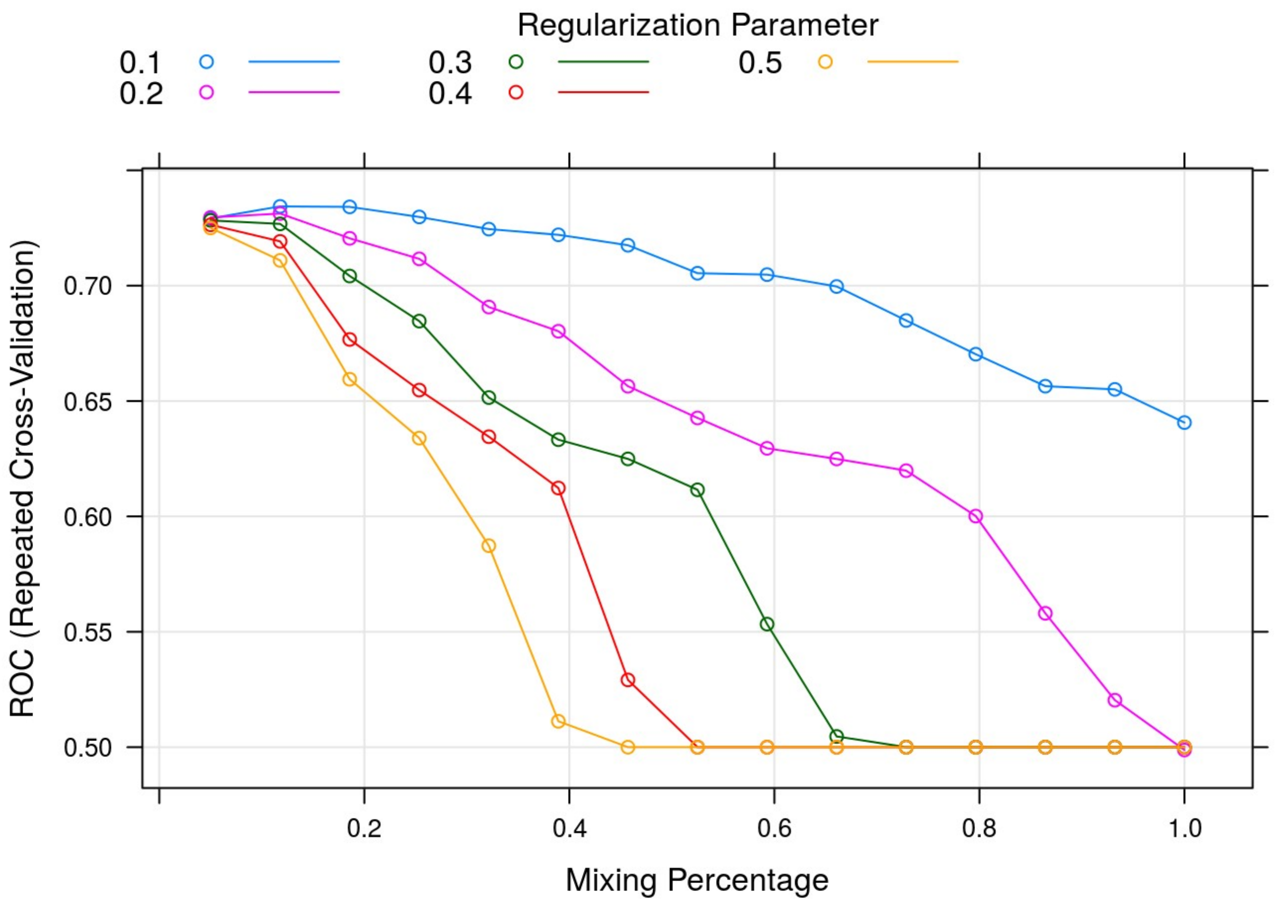


```
train.model.net$bestTune
```

```
##       alpha lambda
## 6 0.1178571    0.1
```

Confusion matrix for Elastic-Net model (with imputed data)

```
pred.cm.net=predict(train.model.net,dataset.x.ts)
cm.net=confusionMatrix(pred.cm.net,dataset.y.ts,positive="Dead")
cm.net
```

```
## Confusion Matrix and Statistics
##
##           Reference
## Prediction Alive Dead
##      Alive  1986   20
##      Dead    639   30
##
##                Accuracy : 0.7536
##                  95% CI : (0.7369, 0.7699)
##     No Information Rate : 0.9813
##     P-Value [Acc > NIR] : 1
##
##                   Kappa : 0.0504
##  Mcnemar's Test P-Value : <2e-16
##
##             Sensitivity : 0.60000
##             Specificity : 0.75657
##          Pos Pred Value : 0.04484
##          Neg Pred Value : 0.99003
##              Prevalence : 0.01869
##          Detection Rate : 0.01121
##    Detection Prevalence : 0.25009
##       Balanced Accuracy : 0.67829
##
##        'Positive' Class : Dead
##
```

3. AUC ROC curves

```
library(pROC)

pred.pls=predict(train.model.pls,dataset.x.ts,type="prob")
pred.rf=predict(train.model.rf,dataset.x.ts,type="prob")
pred.svm=predict(train.model.svm,dataset.x.ts,type="prob")
pred.net=predict(train.model.net,dataset.x.ts,type="prob")
pred.log=predict(train.model.log,dataset.x.ts,type="prob")


roc(predictor=pred.rf$Dead,response=dataset.y.ts,levels=rev(levels(dataset.y.ts)),
ci=T,plot=T,xlab="1 - Specificity")
```

```
##
## Call:
## roc.default(response = dataset.y.ts, predictor = pred.rf$Dead,     levels = rev
(levels(dataset.y.ts)), ci = T, plot = T, xlab = "1 - Specificity")
##
## Data: pred.rf$Dead in 50 controls (dataset.y.ts Dead) > 2625 cases (dataset.y.t
s Alive).
## Area under the curve: 0.7961
## 95% CI: 0.742-0.8502 (DeLong)
```

```
rf.roc=roc(predictor=pred.rf$Dead,response=dataset.y.ts,levels=rev(levels(dataset.
y.ts)),ci=T,plot=T,add=T,col="#0000FF",xlim=c(0,1),main="ROC curves plotting perfo
rmance of different models")
pls.roc=roc(predictor=pred.pls$Dead,response=dataset.y.ts,levels=rev(levels(datase
t.y.ts)),ci=T,plot=T,add=T,col="#CD0000")
log.roc=roc(predictor=pred.log$Dead,response=dataset.y.ts,levels=rev(levels(datase
t.y.ts)),ci=T,plot=T,add=T,col="#000000")
svm.roc=roc(predictor=pred.svm$Dead,response=dataset.y.ts,levels=rev(levels(datase
t.y.ts)),ci=T,plot=T,add=T,col="#FFFF00")
net.roc=roc(predictor=pred.net$Dead,response=dataset.y.ts,levels=rev(levels(datase
t.y.ts)),ci=T,plot=T,add=T,col="#7CFC00")

legend('bottomright',c("AUC Results",
                      paste0("RF Model=",round(rf.roc$auc[1],3)),
                      paste0("PLS-DA Model=",round(pls.roc$auc[1],3)),
                      paste0("Logistic Model=",round(log.roc$auc[1],3)),
                      paste0("Elastic-Net Model=",round(net.roc$auc[1],3)),
                      paste0("Linear Kernel SVM Model=",round(svm.roc$auc[1],3))),
       lty = c(1,1,1,1,1,1),col=c('#FFFFFF','#0000FF','#CD0000','#000000',"#FFFF00
","#7CFC00"))
```

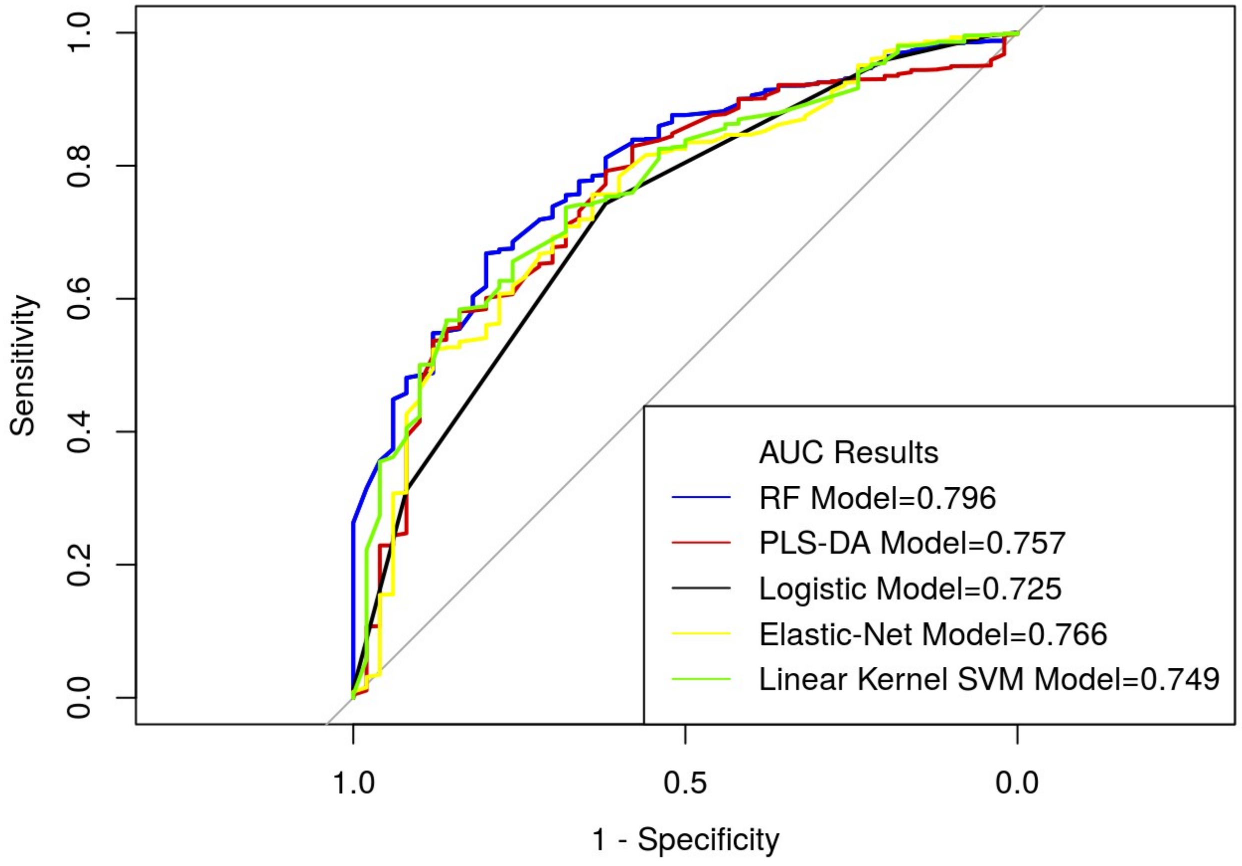

DeLong's test for two correlated ROC curves

```
log.rf.test=roc.test(log.roc,rf.roc)
log.pls.test=roc.test(log.roc,pls.roc)
log.svm.test=roc.test(log.roc,svm.roc)
log.net.test=roc.test(log.roc,net.roc)

p.test.df=data.frame("Model Type"=c("Random Forest",
                                    "PLS-DA",
                                    "Linear Kernel SVM",
                                    "Elastic-Net"),
                      "P-Value"=c(round(log.rf.test$p.value,3),
                                  round(log.pls.test$p.value,3),
                                  round(log.svm.test$p.value,3),
                                  round(log.net.test$p.value,3)))
kable(p.test.df)
```

Model.Type	P.Value
Random Forest	0.008
PLS-DA	0.064
Linear Kernel SVM	0.350
Elastic-Net	0.078

```
auc.ci.df=data.frame("Model Type"=c("Logistic Model",
                                    "Random Forest",
                                    "PLS-DA",
                                    "Linear Kernel SVM",
                                    "Elastic-Net"),
                     "AUC"= c(round(log.roc$auc[1],3),round(rf.roc$auc[1],3),round
(pls.roc$auc[1],3),
                              round(svm.roc$auc[1],3),round(net.roc$auc[1],3)),
                     "95CI"=c(paste0(round(log.roc$ci,3)[1],"-",round(log.roc$ci,3
)[3]),
                              paste0(round(rf.roc$ci,3)[1],"-",round(rf.roc$ci,3)[
3]),
                              paste0(round(pls.roc$ci,3)[1],"-",round(pls.roc$ci,3
)[3]),
                              paste0(round(svm.roc$ci,3)[1],"-",round(svm.roc$ci,3
)[3]),
                              paste0(round(net.roc$ci,3)[1],"-",round(net.roc$ci,3
)[3])))
kable(auc.ci.df)
```

Model.Type	AUC	X95CI
Logistic Model	0.725	0.658-0.792
Random Forest	0.796	0.742-0.85
PLS-DA	0.757	0.691-0.823
Linear Kernel SVM	0.749	0.68-0.817

Model.Type	AUC	X95CI
Elastic-Net	0.766	0.705-0.827

4. Variable Importance of different models

4.1 Variable Importance Plot - PLS-DA

```
vic.pls=varImp(train.model.pls,useModel=T,scale=F)
ggplot(vic.pls)
```

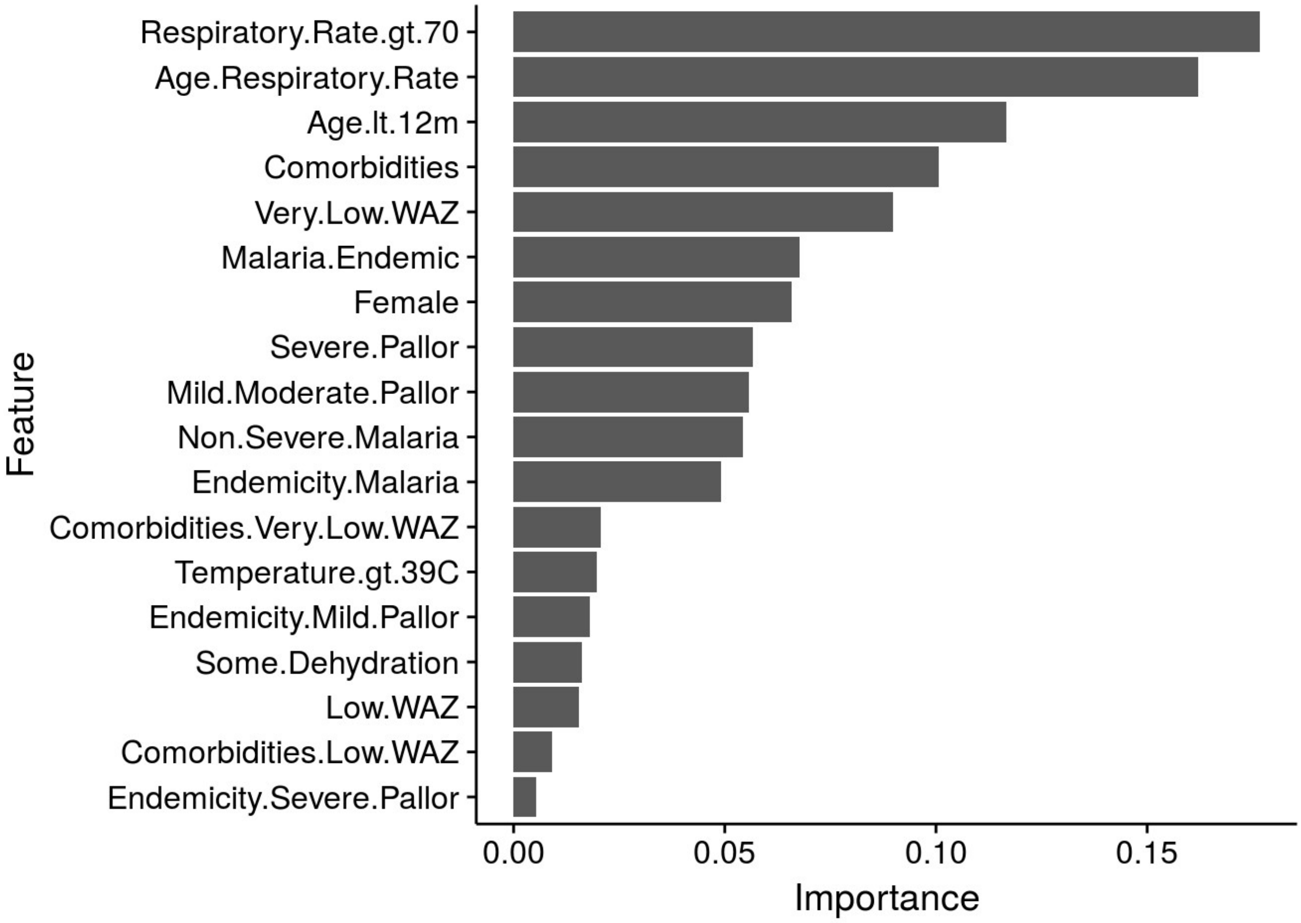

4.2 Variable Importance Plot - Random Forest

```
vic.rf=varImp(train.model.rf,useModel=T,scale=F)
ggplot(vic.rf)
```

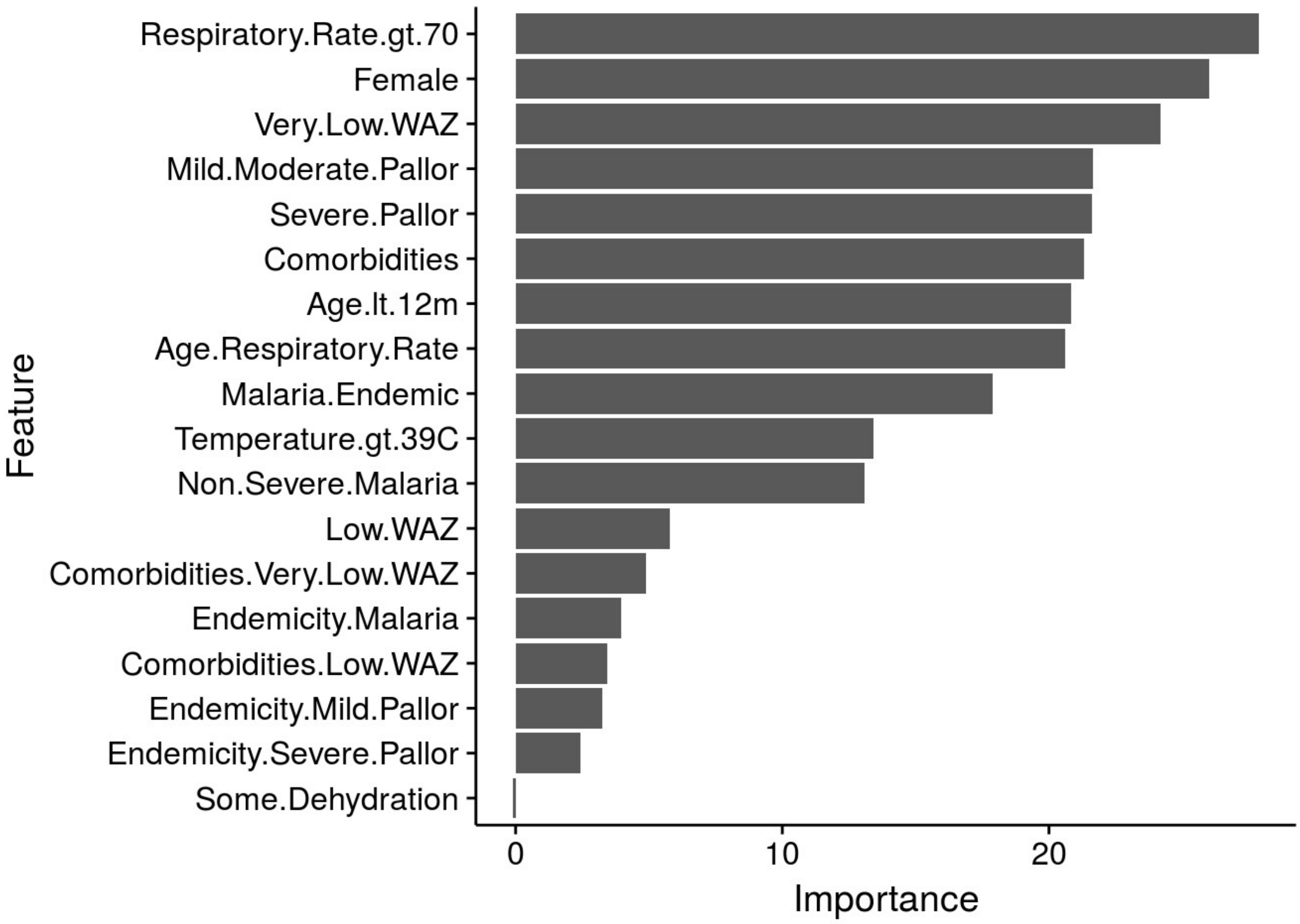

4.3 Variable Importance Plot - SVM

```
vic.svm=varImp(train.model.svm,useModel=T,scale=F)
ggplot(vic.svm)
```

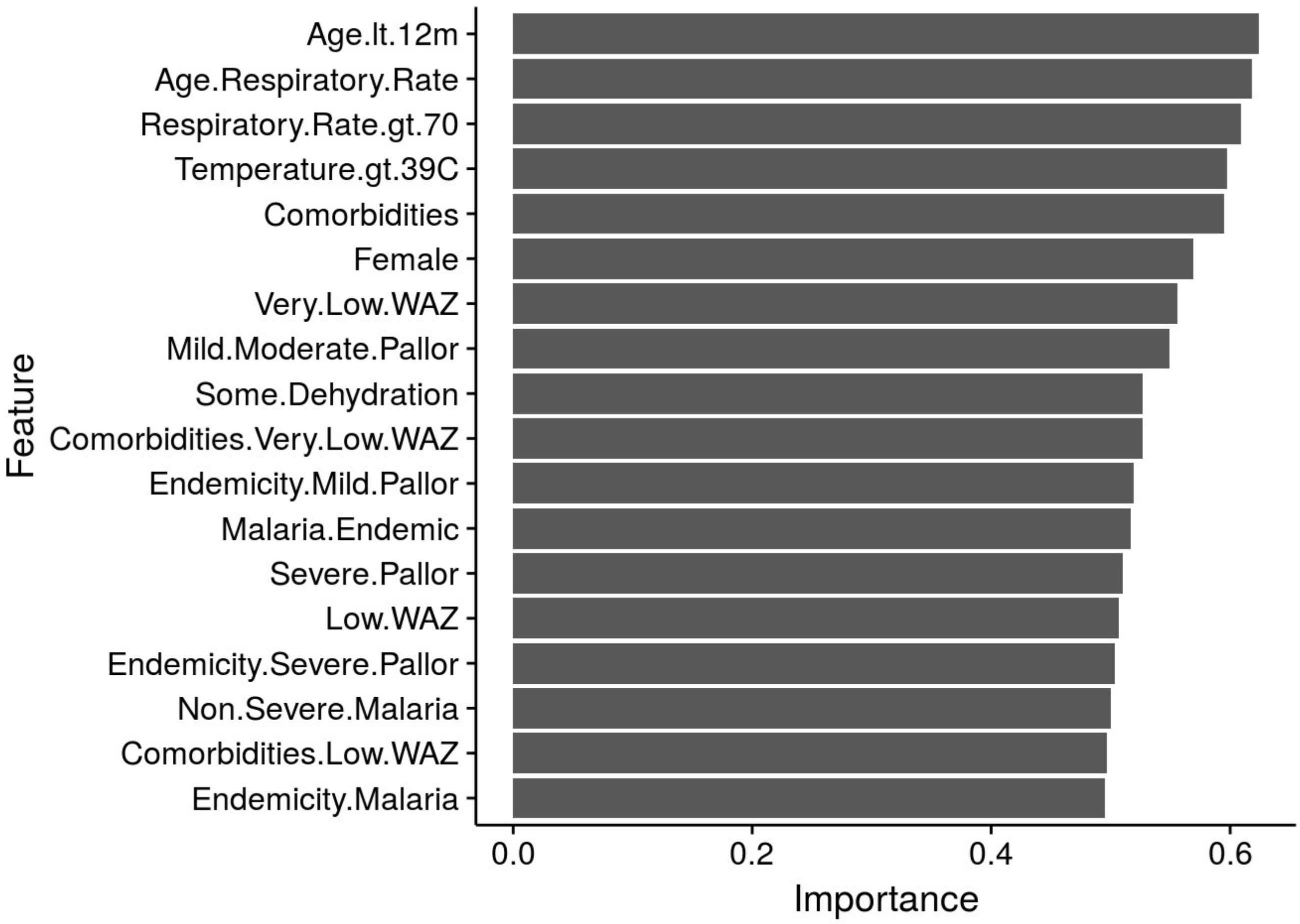

4.4 Variable Importance Plot - Elastic Net

```
vic.net=varImp(train.model.net,useModel=T,scale=F)
ggplot(vic.net)
```

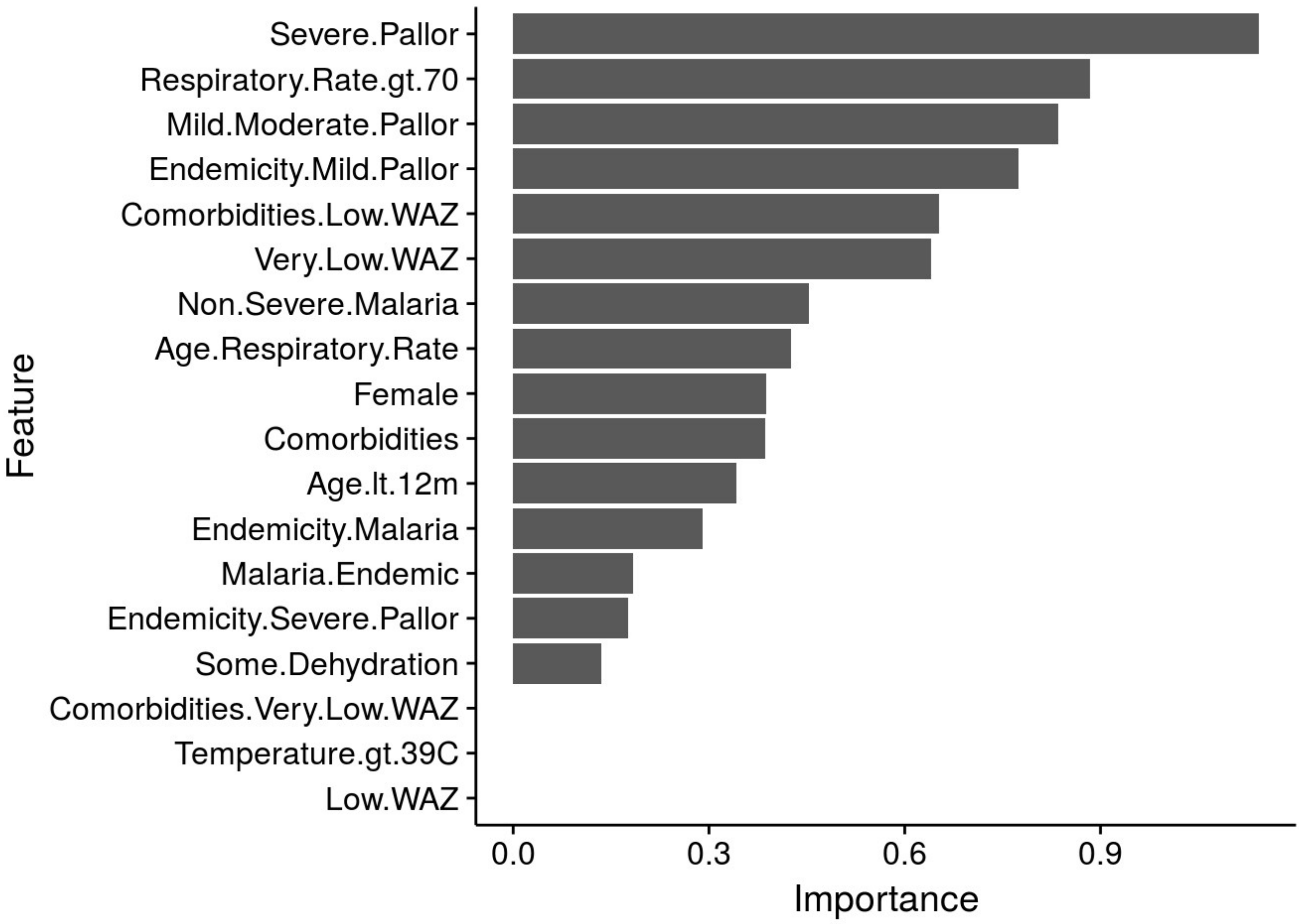

4.5 Variable Importance Plot - Logistic Model

```
vic.log=varImp(train.model.log,useModel=T,scale=F)
ggplot(vic.log)
```

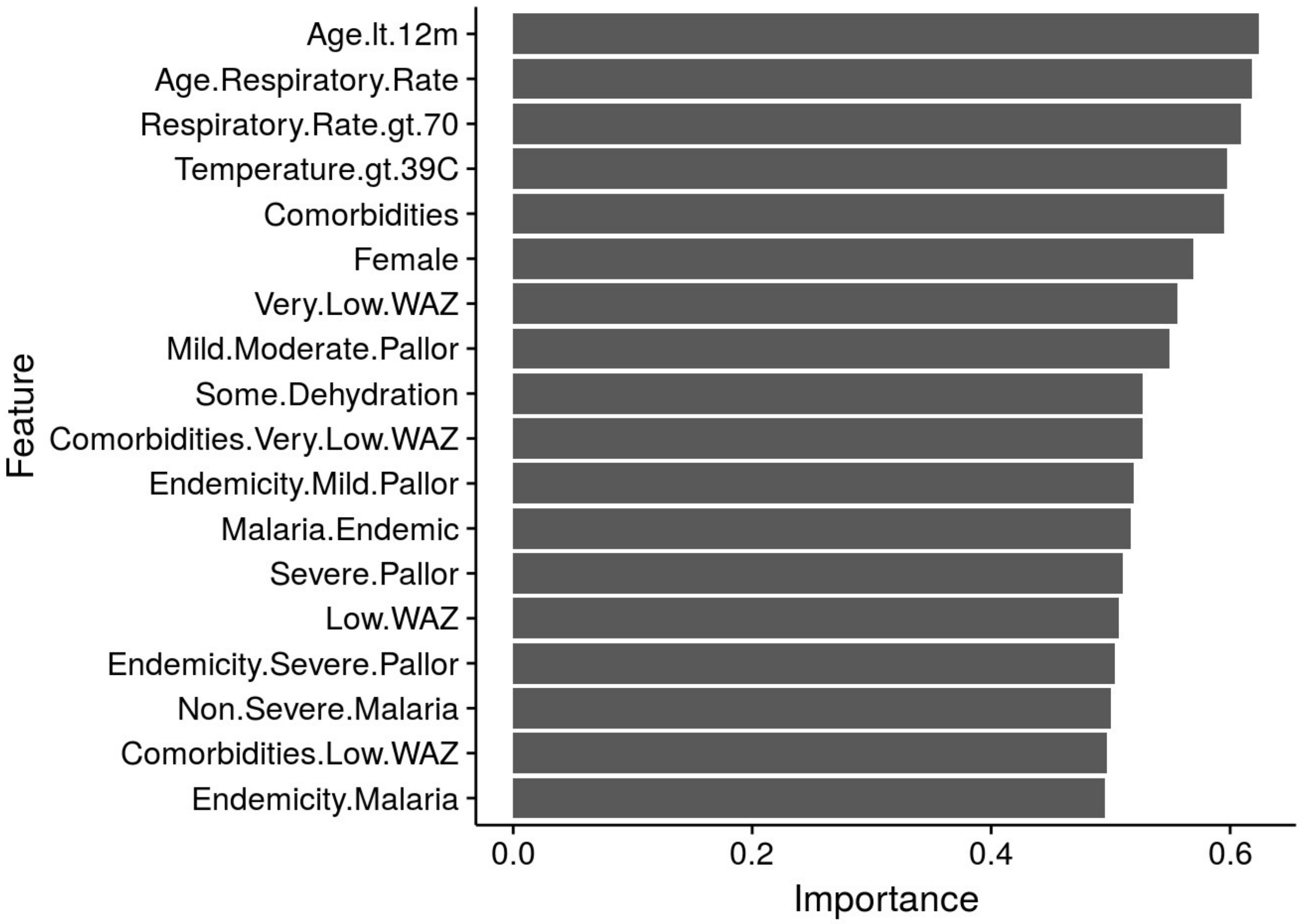

5. Decision curve analysis

```
load(file="pneumonia.test.rda")

pneumonia.org = copy(pneumonia)
pneumonia.org=pneumonia.org[,copy(.SD),.SDcols=c("indrawing","tachypnoea","high.fe
ver","female","mal.end","age.lt.12m","cormobidity","pneum.severity.Non.severe","pn
eum.severity.Severe","pneum.severity.Very.Severe","malaria.Non.severe.malaria","pa
llor.Mild.Moderate","pallor.Severe","dehydration.Some.dehydration","waz.Moderate",
"waz.Severe","malnutrition.Moderate","mortality")]

pneumonia.org[,cormobidity:=cormobidity-1]
pneumonia.org[,cormobidity:=ifelse(cormobidity > 0, 1, 0)]

pneumonia.org[,`WHO None Severe`:=0]
pneumonia.org[pneum.severity.Non.severe==1| pneum.severity.Severe==1,`WHO None Sev
ere`:=1]
pneumonia.org[,pneum.severity.Non.severe:=NULL]
pneumonia.org[,pneum.severity.Severe:=NULL]

setnames(pneumonia.org,
        c("indrawing","age.lt.12m","female","high.fever","cormobidity","pneum.seve
rity.Very.Severe","mal.end","tachypnoea","waz.Moderate","waz.Severe","dehydration.
Some.dehydration","mortality","malaria.Non.severe.malaria","pallor.Mild.Moderate",
"pallor.Severe","malnutrition.Moderate"),
        c("Indrawing","Age < 12m","Female","Temperature ≥39 C","Presence of Cormob
idity","Severe Pneumonia","Malaria Endemic","Respiratory Rate ≥70/min","Low WAZ","
Very Low WAZ","Some Dehydration","Mortality","Non severe Malaria","Mild/Moderate P
allor","Severe Pallor","Moderate Malnutrition"))

pneumonia.dca=copy(pneumonia.org)
pneumonia.dca=pneumonia.dca[,copy(.SD),.SDcols=c("Indrawing","Age < 12m","Female",
"Respiratory Rate ≥70/min","Very Low WAZ","Mild/Moderate Pallor","Presence of Corm
obidity","WHO None Severe","Severe Pneumonia","Mortality")]
pneumonia.dca=pneumonia.dca[complete.cases(pneumonia.dca),copy(.SD)]

set.seed(197)
curr.dec.model=decision_curve(Mortality~ `Severe Pneumonia`,
                              data = pneumonia.dca,
                              thresholds = seq(0, .15, by = .0005),
                              bootstraps = 1000)

set.seed(197)
risk.grp.model <- decision_curve(Mortality ~ `WHO None Severe`+`Respiratory Rate ≥
70/min`+
                                   `Age < 12m` + `Very Low WAZ`,
                                      data = pneumonia.dca,
                                      thresholds = seq(0, .15, by = .0005),
                                      bootstraps = 1000)

plot_decision_curve(list(curr.dec.model,risk.grp.model),
                    curve.names = c("Severe Pneumonia",
                                  "*Age <12m + Resp Rate ≥70\n+Very Low WAZ"),
                    confidence.intervals = FALSE,
                    standardize = FALSE,
                    ylim = c(-0.005, 0.035),
```

```
                    xlim= c(0, 0.15),
                    cost.benefit.axis = FALSE,
                    xaxt="n",
                    main="Decision Curve Analysis Graph")
```

```
## Note: When multiple decision curves are plotted, decision curves for 'All' are
calculated using the first DecisionCurve in the list provided.
```

```
axis(1,at = seq(0, 0.15, by = 0.01),las=2)
abline(h=seq(0, 0.15, by = 0.005), v=seq(0, 0.15, by = 0.005), col="gray", lty=3)
```

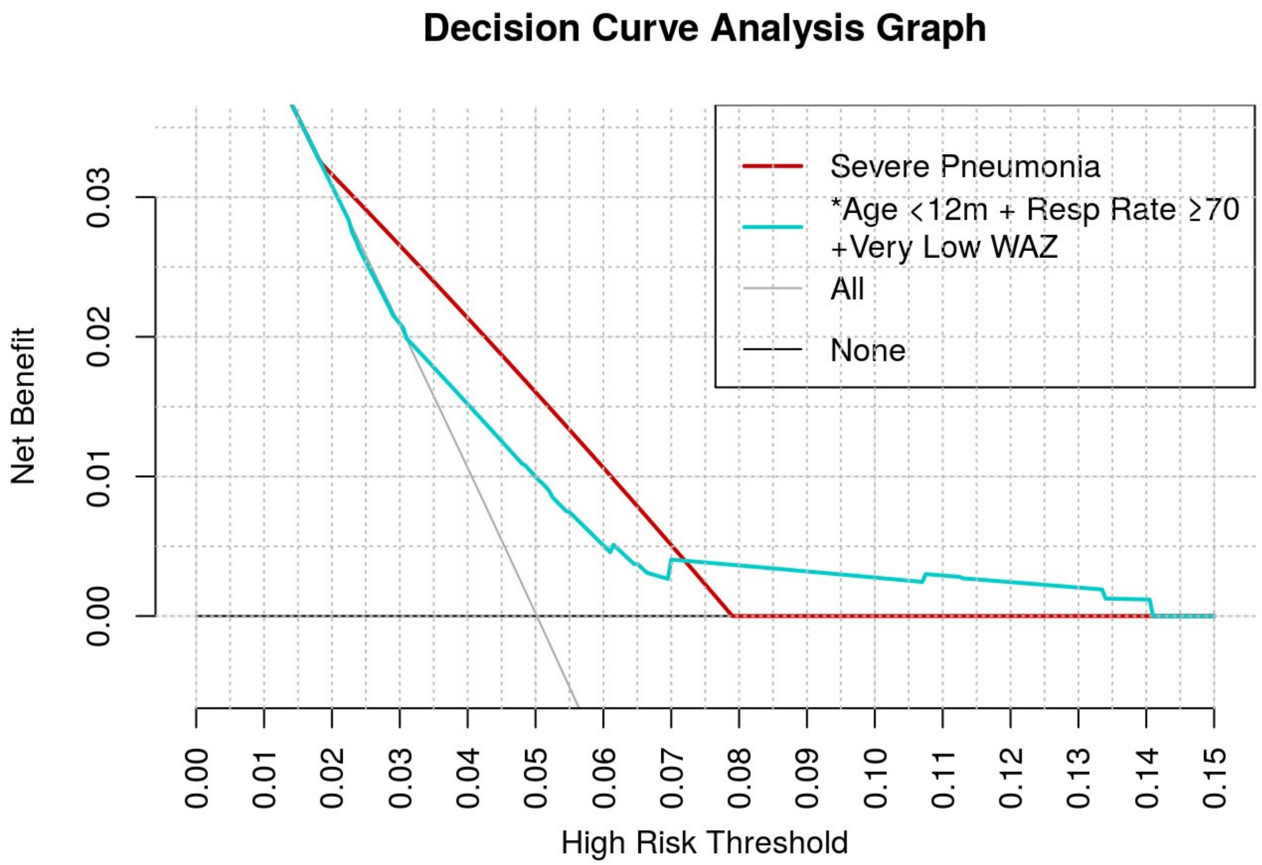

Decision impact

```
#plot the clinical impact
plot_clinical_impact(risk.grp.model, xlim = c(0, 0.12),col = c("orange", "red"),
                     population.size = 100,
                     cost.benefit.axis = FALSE,
                     main="Clinical Impact Graph")
```

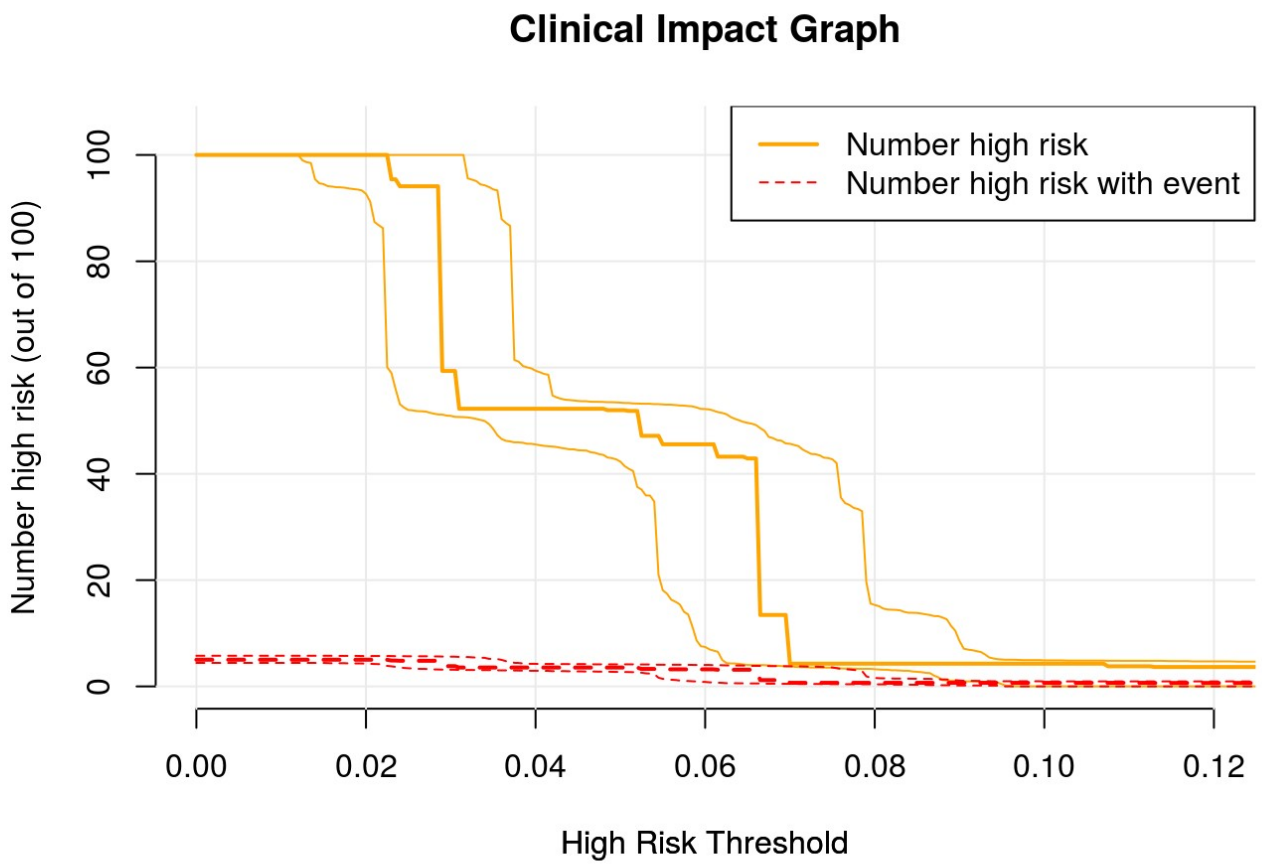

6. Sensitivity analysis

6.1 Based on different classification criterion

6.1.1 Penicillin Monotherapy

```
pneumonia= pneumonia.copy[,copy(.SD)]
suppressMessages({

  pneumonia[,Age.Respiratory.Rate:=ifelse(!is.na(Age.lt.12m) & !is.na(Respiratory.
Rate.gt.70),0,NA_integer_)]
  pneumonia[Age.Respiratory.Rate==0 & Age.lt.12m==1 & Respiratory.Rate.gt.70==1,Ag
e.Respiratory.Rate:=1]

  pneumonia[,Comorbidities.Low.WAZ:=ifelse(!is.na(Comorbidities) & !is.na(Low.WAZ)
,0,NA_integer_)]
  pneumonia[Comorbidities.Low.WAZ==0 & Comorbidities==1 & Low.WAZ==1,Comorbidities
.Low.WAZ:=1]

  pneumonia[,Comorbidities.Very.Low.WAZ:=ifelse(!is.na(Comorbidities) & !is.na(Ver
y.Low.WAZ),0,NA_integer_)]
  pneumonia[Comorbidities.Very.Low.WAZ==0 & Comorbidities==1 & Very.Low.WAZ==1,Com
orbidities.Very.Low.WAZ:=1]

  pneumonia[,Endemicity.Malaria:=ifelse(!is.na(Malaria.Endemic) & !is.na(Non.Sever
e.Malaria),0,NA_integer_)]
  pneumonia[Endemicity.Malaria==0 & Malaria.Endemic==1 & Non.Severe.Malaria==1,End
emicity.Malaria:=1]

  pneumonia[,Endemicity.Mild.Pallor:=ifelse(!is.na(Malaria.Endemic) & !is.na(Mild.
Moderate.Pallor),0,NA_integer_)]
  pneumonia[Endemicity.Mild.Pallor==0 & Malaria.Endemic==1 & Mild.Moderate.Pallor=
=1,Endemicity.Mild.Pallor:=1]

  pneumonia[,Endemicity.Severe.Pallor:=ifelse(!is.na(Malaria.Endemic) & !is.na(Sev
ere.Pallor),0,NA_integer_)]
  pneumonia[Endemicity.Severe.Pallor==0 & Malaria.Endemic==1 & Severe.Pallor==1,En
demicity.Severe.Pallor:=1]

  setcolorder(pneumonia,c("Age.lt.12m","Female","Respiratory.Rate.gt.70","Temperat
ure.gt.39C","Severe.Pneumonia","Mild.Moderate.Pallor","Severe.Pallor","Some.Dehydr
ation","Low.WAZ","Very.Low.WAZ","Malaria.Endemic","Non.Severe.Malaria","Comorbidit
ies","Severity.Treatment","Clinician.Diagnosis","None.Severe","Age.Respiratory.Rat
e","Comorbidities.Very.Low.WAZ","Comorbidities.Low.WAZ","Endemicity.Malaria","Ende
micity.Mild.Pallor","Endemicity.Severe.Pallor","Mortality"))
})

#Based on Penicillin Monotherapy
pneumonia.pres=pneumonia[Severity.Treatment==1,copy(.SD)]
pneumonia.pres[,Clinician.Diagnosis:=NULL]
pneumonia.pres[,Severity.Treatment:=NULL]
pneumonia.pres[,None.Severe:=NULL]
pneumonia.pres[,Severe.Pneumonia:=NULL]

dataset=pneumonia.pres[!is.na(Mortality),copy(.SD)]
dataset$Mortality=factor(dataset$Mortality,levels=c(0,1),labels=c("Alive","Dead"))

paste0(round(prop.table(table(dataset$Mortality))*100,2)," %")
```

```
## [1] "99.26 %" "0.74 %"
```

```
dataset=as.data.frame(dataset)

set.seed(197)
train.ids=sample(seq(nrow(dataset)),round(nrow(dataset)*0.67),replace=F)

col.len=length(dataset)

dataset.x=dataset[,1:(col.len-1)]
dataset.y=dataset[,col.len]

dataset.x.tr.pres=dataset.x[train.ids,]
dataset.y.tr.pres=dataset.y[train.ids]

dataset.x.ts.pres=dataset.x[-train.ids,]
dataset.y.ts.pres=dataset.y[-train.ids]

tune.len=length(dataset.x.tr.pres)

set.seed(197)
train.model.pls.pres=train(x=dataset.x.tr.pres
                           ,y=dataset.y.tr.pres
                           ,method="pls"
                           ,trControl=ctrl
                           ,tuneLength = tune.len
                           ,importance=T
                           ,probMethod="Bayes"
                           ,metric="ROC"
                           ,preProc=c(method="bagImpute")
)
```

Confusion matrix for Penicillin Monotherapy model (with imputed data)

```
pred.pls.pres=predict(train.model.pls.pres,dataset.x.ts.pres)
cm.pls.pres=confusionMatrix(pred.pls.pres,dataset.y.ts.pres,positive="Dead")
cm.pls.pres
```

```
## Confusion Matrix and Statistics
##
##           Reference
## Prediction Alive Dead
##      Alive   983    6
##      Dead    212    3
##
##                Accuracy : 0.8189
##                  95% CI : (0.796, 0.8403)
##     No Information Rate : 0.9925
##     P-Value [Acc > NIR] : 1
##
##                   Kappa : 0.0126
##  Mcnemar's Test P-Value : <2e-16
##
##             Sensitivity : 0.333333
##             Specificity : 0.822594
##          Pos Pred Value : 0.013953
##          Neg Pred Value : 0.993933
##              Prevalence : 0.007475
##          Detection Rate : 0.002492
##    Detection Prevalence : 0.178571
##       Balanced Accuracy : 0.577964
##
##        'Positive' Class : Dead
##
```

6.1.2 Clinician Diagnosis

```
#Based on Clinician Diagnosis
pneumonia.cd=pneumonia[Clinician.Diagnosis==1,copy(.SD)]
pneumonia.cd[,Clinician.Diagnosis:=NULL]
pneumonia.cd[,Severity.Treatment:=NULL]
pneumonia.cd[,None.Severe:=NULL]
pneumonia.cd[,Severe.Pneumonia:=NULL]

dataset=pneumonia.cd[!is.na(Mortality),copy(.SD)]
dataset$Mortality=factor(dataset$Mortality,levels=c(0,1),labels=c("Alive","Dead"))

paste0(round(prop.table(table(dataset$Mortality))*100,2)," %")
```

```
## [1] "97.64 %" "2.36 %"
```

```
dataset.comp.cd=copy(dataset)
dataset=as.data.frame(dataset)

set.seed(197)
train.ids=sample(seq(nrow(dataset)),round(nrow(dataset)*0.67),replace=F)

col.len=length(dataset)

dataset.x=dataset[,1:(col.len-1)]
dataset.y=dataset[,col.len]

dataset.x.tr.cd=dataset.x[train.ids,]
dataset.y.tr.cd=dataset.y[train.ids]

dataset.x.ts.cd=dataset.x[-train.ids,]
dataset.y.ts.cd=dataset.y[-train.ids]

tune.len=length(dataset.x.tr.cd)

set.seed(197)
train.model.pls.cd=train(x=dataset.x.tr.cd
                         ,y=dataset.y.tr.cd
                         ,method="pls"
                         ,trControl=ctrl
                         ,tuneLength = tune.len
                         ,importance=T
                         ,probMethod="Bayes"
                         ,metric="ROC"
                         ,preProc=c(method="bagImpute")
)
```

Confusion matrix for Clinician Diagnosis model (with imputed data)

```
pred.pls.cd=predict(train.model.pls.cd,dataset.x.ts.cd)
cm.pls.cd=confusionMatrix(pred.pls.cd,dataset.y.ts.cd,positive="Dead")
cm.pls.cd
```

```
## Confusion Matrix and Statistics
##
##           Reference
## Prediction Alive Dead
##      Alive  2565   32
##      Dead    704   49
##
##                Accuracy : 0.7803
##                  95% CI : (0.7659, 0.7942)
##     No Information Rate : 0.9758
##     P-Value [Acc > NIR] : 1
##
##                   Kappa : 0.0772
##  Mcnemar's Test P-Value : <2e-16
##
##             Sensitivity : 0.60494
##             Specificity : 0.78464
##          Pos Pred Value : 0.06507
##          Neg Pred Value : 0.98768
##              Prevalence : 0.02418
##          Detection Rate : 0.01463
##    Detection Prevalence : 0.22478
##       Balanced Accuracy : 0.69479
##
##        'Positive' Class : Dead
##
```

6.1.3 Ideal population

```
#Based on Ideal Population
pneumonia.idl=pneumonia[Severity.Treatment==1 & Clinician.Diagnosis==1 & None.Seve
re==1,copy(.SD)]
pneumonia.idl[,Clinician.Diagnosis:=NULL]
pneumonia.idl[,Severity.Treatment:=NULL]
pneumonia.idl[,None.Severe:=NULL]
pneumonia.idl[,Severe.Pneumonia:=NULL]

dataset=pneumonia.idl[!is.na(Mortality),copy(.SD)]
dataset$Mortality=factor(dataset$Mortality,levels=c(0,1),labels=c("Alive","Dead"))

paste0(round(prop.table(table(dataset$Mortality))*100,2)," %")
```

```
## [1] "99.27 %" "0.73 %"
```

```
dataset=as.data.frame(dataset)

set.seed(197)
train.ids=sample(seq(nrow(dataset)),round(nrow(dataset)*0.67),replace=F)

col.len=length(dataset)

dataset.x=dataset[,1:(col.len-1)]
dataset.y=dataset[,col.len]

dataset.x.tr.idl=dataset.x[train.ids,]
dataset.y.tr.idl=dataset.y[train.ids]

dataset.x.ts.idl=dataset.x[-train.ids,]
dataset.y.ts.idl=dataset.y[-train.ids]

tune.len=length(dataset.x.tr.idl)

set.seed(197)
train.model.pls.idl=train(x=dataset.x.tr.idl
                          ,y=dataset.y.tr.idl
                          ,method="pls"
                          ,trControl=ctrl
                          ,tuneLength = tune.len
                          ,importance=T
                          ,probMethod="Bayes"
                          ,metric="ROC"
                          ,preProc=c(method="bagImpute")
)
```

Confusion matrix for Ideal population model (with imputed data)

```
pred.pls.idl=predict(train.model.pls.idl,dataset.x.ts.idl)
cm.pls.idl=confusionMatrix(pred.pls.idl,dataset.y.ts.idl,positive="Dead")
cm.pls.idl
```

```
## Confusion Matrix and Statistics
##
##           Reference
## Prediction Alive Dead
##      Alive   706    3
##      Dead    143    2
##
##                Accuracy : 0.829
##                  95% CI : (0.8021, 0.8537)
##     No Information Rate : 0.9941
##     P-Value [Acc > NIR] : 1
##
##                   Kappa : 0.0155
##  Mcnemar's Test P-Value : <2e-16
##
##             Sensitivity : 0.400000
##             Specificity : 0.831567
##          Pos Pred Value : 0.013793
##          Neg Pred Value : 0.995769
##              Prevalence : 0.005855
##          Detection Rate : 0.002342
##    Detection Prevalence : 0.169789
##       Balanced Accuracy : 0.615783
##
##        'Positive' Class : Dead
##
```

Getting the AUC and DeLong’s test for the ROC curves

```
pred.cd=predict(train.model.pls.cd,dataset.x.ts.cd,type="prob")
pred.press=predict(train.model.pls.pres,dataset.x.ts.pres,type="prob")
pred.idl=predict(train.model.pls.idl,dataset.x.ts.idl,type="prob")

roc(predictor=pred.pls$Dead,response=dataset.y.ts,levels=rev(levels(dataset.y.ts))
,ci=T,plot=T,xlab="1 - Specificity")
```

```
##
## Call:
## roc.default(response = dataset.y.ts, predictor = pred.pls$Dead,     levels = re
v(levels(dataset.y.ts)), ci = T, plot = T, xlab = "1 - Specificity")
##
## Data: pred.pls$Dead in 50 controls (dataset.y.ts Dead) > 2625 cases (dataset.y.
ts Alive).
## Area under the curve: 0.757
## 95% CI: 0.6909-0.8231 (DeLong)
```

```
pls.roc=roc(predictor=pred.pls$Dead,response=dataset.y.ts,levels=rev(levels(datase
t.y.ts)),ci=T,plot=T,add=T,col="#0000FF",xlim=c(0,1)
            ,main="ROC curves plotting performance of pneumonia criteria")
pls.cd.roc=roc(predictor=pred.cd$Dead,response=dataset.y.ts.cd,levels=rev(levels(d
ataset.y.ts.cd)),ci=T,plot=T,add=T,col="#CD0000")
pls.press.roc=roc(predictor=pred.press$Dead,response=dataset.y.ts.pres,levels=rev(
levels(dataset.y.ts.pres)),ci=T,plot=T,add=T,col="#000000")
pls.idl.roc=roc(predictor=pred.idl$Dead,response=dataset.y.ts.idl,levels=rev(level
s(dataset.y.ts.idl)),ci=T,plot=T,add=T,col="#FFFF00")

legend('bottomright',c("AUC Results",
                      paste0("WHO criteria=",round(pls.roc$auc[1],3)),
                      paste0("Clinician Criteria=",round(pls.cd.roc$auc[1],3)),
                      paste0("Penicillin Criteria=",round(pls.press.roc$auc[1],3))
,
                      paste0("Ideal Criteria=",round(pls.idl.roc$auc[1],3))),
       lty = c(1,1,1,1,1),col=c('#FFFFFF','#0000FF','#CD0000','#000000',"#FFFF00")
)
```

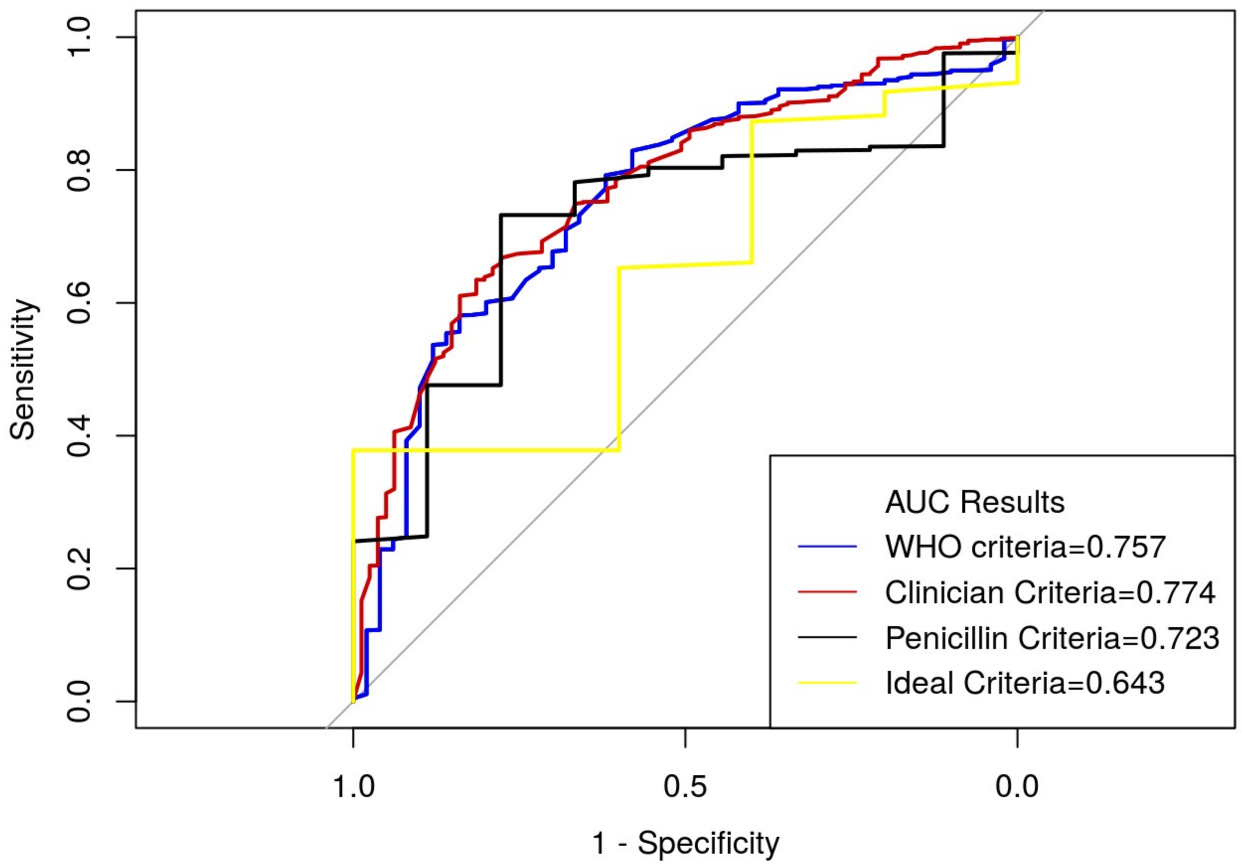

```
who.cd.test=roc.test(pls.roc,pls.cd.roc)
who.press.test=roc.test(pls.roc,pls.press.roc)
who.idl.test=roc.test(pls.roc,pls.idl.roc)

p.test.sens.df=data.frame("Pneumonia Classification Criteria"=c("Clinician Diagnos
is Criteria","Penicillin Monotherapy Criteria","Ideal population"),"P-Value"=c(rou
nd(who.cd.test$p.value,3),round(who.press.test$p.value,3),round(who.idl.test$p.val
ue,3)))

kable(p.test.sens.df)
```

Pneumonia.Classification.Criteria	P.Value
Clinician Diagnosis Criteria	0.691
Penicillin Monotherapy Criteria	0.679
Ideal population	0.352

```
auc.crit.df=data.frame("Model Type"=c("WHO criteria",
                                    "Clinician Criteria",
                                    "Penicillin Criteria",
                                    "Ideal Criteria"),
                     "AUC"= c(round(pls.roc$auc[1],3),round(pls.cd.roc$auc[1],3),r
ound(pls.press.roc$auc[1],3),
                              round(pls.idl.roc$auc[1],3)),
                     "95% CI"=c(paste0(round(pls.roc$ci,3)[1],"-",round(pls.roc$ci
,3)[3]),
                              paste0(round(pls.cd.roc$ci,3)[1],"-",round(pls.cd.ro
c$ci,3)[3]),
                              paste0(round(pls.press.roc$ci,3)[1],"-",round(pls.pr
ess.roc$ci,3)[3]),
                              paste0(round(pls.idl.roc$ci,3)[1],"-",round(pls.idl.
roc$ci,3)[3])))
kable(auc.crit.df)
```

Model.Type	AUC	X95..CI
WHO criteria	0.757	0.691-0.823
Clinician Criteria	0.774	0.726-0.821
Penicillin Criteria	0.723	0.576-0.87
Ideal Criteria	0.643	0.412-0.874

Compare variable imporance for the different classification criterion

```
vic=varImp(train.model.pls,useModel=T,scale=F)
vic.cd=varImp(train.model.pls.cd,useModel=T,scale=F)
vic.tr=varImp(train.model.pls.pres,useModel=T,scale=F)
vic.idl=varImp(train.model.pls.idl,useModel=T,scale=F)

vi=c("vic","vic.cd","vic.tr","vic.idl")
for(i in vi){
  cmd=paste0("row.names(",i,"$importance)[row.names(",i,"$importance)=='Age.lt.12m
']='Age < 12m'")
  eval(parse(text=cmd))
  cmd=paste0("row.names(",i,"$importance)[row.names(",i,"$importance)=='Respirator
y.Rate.gt.70']='Respiratory.Rate > 70'")
  eval(parse(text=cmd))
  cmd=paste0("row.names(",i,"$importance)[row.names(",i,"$importance)=='Temperatur
e.gt.39C']='Temperature > 39C'")
  eval(parse(text=cmd))
}


library(cowplot)
```

```
ggdraw()+
  draw_plot(ggplot(vic)+ggtitle("WHO guidelines")+theme(axis.text=element_text(siz
e=9)),x=0,y=0.75,width=1,height=.25) +
  draw_plot(ggplot(vic.cd)+ggtitle("Clinician Diagnosis")+theme(axis.text=element_
text(size=9)),x=0,y=.5,width=1,height=.25) +
  draw_plot(ggplot(vic.tr)+ggtitle("Penicillin Monotherapy")+theme(axis.text=eleme
nt_text(size=9)),x=0,y=.25,width=1,height=.25) +
  draw_plot(ggplot(vic.idl)+ggtitle("Ideal Population")+theme(axis.text=element_te
xt(size=9)),x=0,y=0,width=1,height=.25)
```

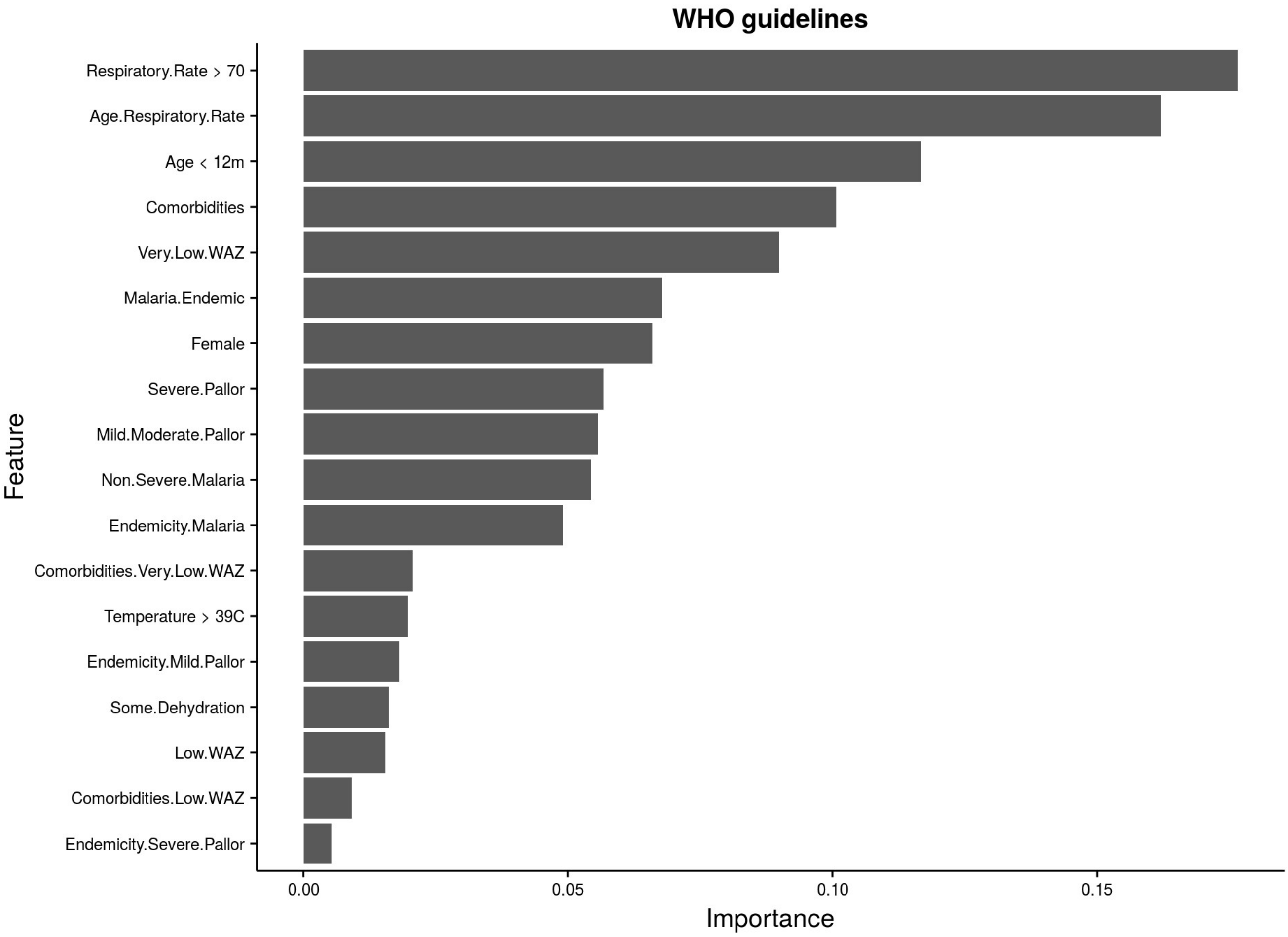
WHO guidelines
Respiratory.Rate > 70
Age.Respiratory.Rate
Age < 12m
Comorbidities
Very.Low.WAZ
Malaria.Endemic
Female
Severe.Pallor
Mild.Moderate.Pallor
Non.Severe.Malaria
Endemicity.Malaria
Comorbidities.Very.Low.WAZ
Temperature > 39C
Endemicity.Mild.Pallor
Some.Dehydration
Low.WAZ
Comorbidities.Low.WAZ
Endemicity.Severe.Pallor
Feature
0.00
0.05
0.10
0.15
Importance

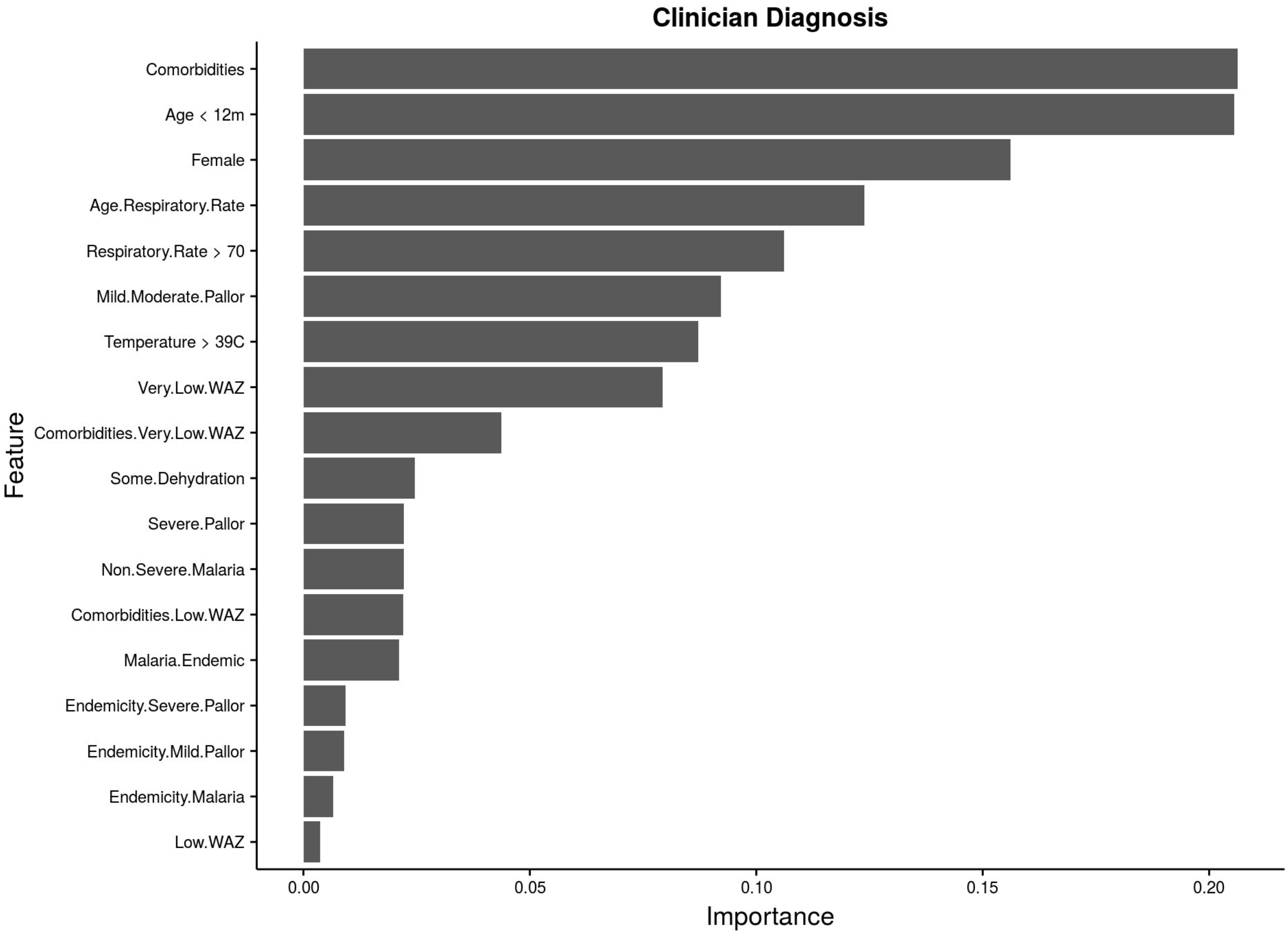
Clinician Diagnosis
Comorbidities
Age < 12m
Female
Age.Respiratory.Rate
Respiratory.Rate > 70
Mild.Moderate.Pallor
Temperature > 39C
Very.Low.WAZ
Comorbidities.Very.Low.WAZ
Some.Dehydration
Severe.Pallor
Non.Severe.Malaria
Comorbidities.Low.WAZ
Malaria.Endemic
Endemicity.Severe.Pallor
Endemicity.Mild.Pallor
Endemicity.Malaria
Low.WAZ
Feature
0.00
0.05
0.10
0.15
0.20
Importance

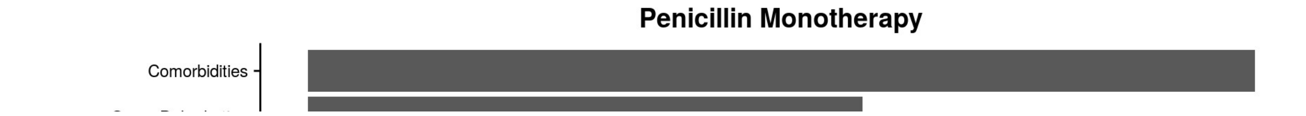
Penicillin Monotherapy
Comorbidities

6.2 Comparing results with complete case analysis

6.2.1 Generate models’ confusion matrix

```
load(file="pneumonia.rda")

pneumonia=pneumonia[,copy(.SD),.SDcols=c("age.lt.12m","female","tachypnoea","high.
fever","pneum.severity.Non.severe","pneum.severity.Severe","pneum.severity.Very.Se
vere","pallor.Mild.Moderate","pallor.Severe","dehydration.Some.dehydration","waz.M
oderate","waz.Severe","mal.end","malaria.Non.severe.malaria","cormobidity","mortal
ity","ns.pneum","clinician.diagnosis")]

pneumonia[,`None Severe`:=0]
pneumonia[pneum.severity.Non.severe==1 | pneum.severity.Severe==1,`None Severe`:=1
]
pneumonia[,pneum.severity.Non.severe:=NULL]
pneumonia[,pneum.severity.Severe:=NULL]
pneumonia[!is.na(clinician.diagnosis),clinician.diagnosis:=ifelse(clinician.diagno
sis==1,NA_real_,
ifelse(clinician.diagnosis==2,1,
ifelse(clinician.diagnosis==3,1,0)))]

pneumonia = pneumonia[,lapply(.SD,as.character)]
pneumonia = pneumonia[,lapply(.SD,as.numeric)]

setnames(pneumonia,
c("age.lt.12m","female","high.fever","pneum.severity.Very.Severe","tachypnoea","wa
z.Moderate","waz.Severe","dehydration.Some.dehydration","mortality","pallor.Mild.M
oderate","pallor.Severe","ns.pneum","clinician.diagnosis","None Severe","mal.end",
"malaria.Non.severe.malaria", "cormobidity"),
c("Age.lt.12m","Female","Temperature.gt.39C","Severe.Pneumonia","Respiratory.Rate.
gt.70","Low.WAZ","Very.Low.WAZ","Some.Dehydration","Mortality","Mild.Moderate.Pall
or","Severe.Pallor","Severity.Treatment","Clinician.Diagnosis","None.Severe","Mala
ria.Endemic","Non.Severe.Malaria","Comorbidities"))

suppressMessages({

  pneumonia[,Age.Respiratory.Rate:=ifelse(!is.na(Age.lt.12m) & !is.na(Respiratory.
Rate.gt.70),0,NA_integer_)]
  pneumonia[Age.Respiratory.Rate==0 & Age.lt.12m==1 & Respiratory.Rate.gt.70==1,Ag
e.Respiratory.Rate:=1]

  pneumonia[,Comorbidities.Low.WAZ:=ifelse(!is.na(Comorbidities) & !is.na(Low.WAZ)
,0,NA_integer_)]
  pneumonia[Comorbidities.Low.WAZ==0 & Comorbidities==1 & Low.WAZ==1,Comorbidities
.Low.WAZ:=1]

  pneumonia[,Comorbidities.Very.Low.WAZ:=ifelse(!is.na(Comorbidities) & !is.na(Ver
y.Low.WAZ),0,NA_integer_)]
  pneumonia[Comorbidities.Very.Low.WAZ==0 & Comorbidities==1 & Very.Low.WAZ==1,Com
orbidities.Very.Low.WAZ:=1]

  pneumonia[,Endemicity.Malaria:=ifelse(!is.na(Malaria.Endemic) & !is.na(Non.Sever
e.Malaria),0,NA_integer_)]
  pneumonia[Endemicity.Malaria==0 & Malaria.Endemic==1 & Non.Severe.Malaria==1,End
emicity.Malaria:=1]

  pneumonia[,Endemicity.Mild.Pallor:=ifelse(!is.na(Malaria.Endemic) & !is.na(Mild.
```

```
Moderate.Pallor),0,NA_integer_)]
  pneumonia[Endemicity.Mild.Pallor==0 & Malaria.Endemic==1 & Mild.Moderate.Pallor=
=1,Endemicity.Mild.Pallor:=1]

  pneumonia[,Endemicity.Severe.Pallor:=ifelse(!is.na(Malaria.Endemic) & !is.na(Sev
ere.Pallor),0,NA_integer_)]
  pneumonia[Endemicity.Severe.Pallor==0 & Malaria.Endemic==1 & Severe.Pallor==1,En
demicity.Severe.Pallor:=1]

  setcolorder(pneumonia,c("Age.lt.12m","Female","Respiratory.Rate.gt.70","Temperat
ure.gt.39C","Severe.Pneumonia","Mild.Moderate.Pallor","Severe.Pallor","Some.Dehydr
ation","Low.WAZ","Very.Low.WAZ","Malaria.Endemic","Non.Severe.Malaria","Comorbidit
ies","Severity.Treatment","Clinician.Diagnosis","None.Severe","Age.Respiratory.Rat
e","Comorbidities.Very.Low.WAZ","Comorbidities.Low.WAZ","Endemicity.Malaria","Ende
micity.Mild.Pallor","Endemicity.Severe.Pallor","Mortality"))
})

#Based on WHO guidelines
pneumonia.ns=pneumonia[None.Severe==1,copy(.SD)]
pneumonia.ns[,None.Severe:=NULL]
pneumonia.ns[,Clinician.Diagnosis:=NULL]
pneumonia.ns[,Severity.Treatment:=NULL]
pneumonia.ns[,Severe.Pneumonia:=NULL]


dataset=pneumonia.ns[!is.na(Mortality),copy(.SD)]
dataset$Mortality=factor(dataset$Mortality,levels=c(0,1),labels=c("Alive","Dead"))

dataset.tr=copy(dataset)

##For complete cases
dataset.comp=copy(dataset.tr)
dataset.comp=dataset.comp[complete.cases(dataset.comp),copy(.SD)]
dataset.comp=as.data.frame(dataset.comp)

set.seed(197)
train.ids.comp=sample(seq(nrow(dataset.comp)),round(nrow(dataset.comp)*0.67),repla
ce=F)

dataset.x.comp=dataset.comp[,1:(col.len-1)]
dataset.y.comp=dataset.comp[,col.len]

dataset.x.tr.comp=dataset.x.comp[train.ids.comp,]
dataset.y.tr.comp=dataset.y.comp[train.ids.comp]

dataset.x.ts.comp=dataset.x.comp[-train.ids.comp,]
dataset.y.ts.comp=dataset.y.comp[-train.ids.comp]

tune.len=length(dataset.x.tr.comp)
```

PLS-DA model

```
set.seed(197)
train.model.pls.comp=train(x=dataset.x.tr.comp
                          ,y=dataset.y.tr.comp
                          ,method="pls"
                          ,trControl=ctrl
                          ,tuneLength = tune.len
                          ,importance=T
                          ,probMethod="Bayes"
                          ,metric="ROC"
)
```

Confusion matrix for PLS-DA model (complete cases)

```
pred.cm.who.comp=predict(train.model.pls.comp,dataset.x.ts.comp)
cm.who.comp=confusionMatrix(pred.cm.who.comp,dataset.y.ts.comp,positive="Dead")
cm.who.comp
```

```
## Confusion Matrix and Statistics
##
##           Reference
## Prediction Alive Dead
##      Alive  1385   12
##      Dead    407   13
##
##                Accuracy : 0.7694
##                  95% CI : (0.7493, 0.7886)
##     No Information Rate : 0.9862
##     P-Value [Acc > NIR] : 1
##
##                   Kappa : 0.0333
##  Mcnemar's Test P-Value : <2e-16
##
##             Sensitivity : 0.520000
##             Specificity : 0.772879
##          Pos Pred Value : 0.030952
##          Neg Pred Value : 0.991410
##              Prevalence : 0.013759
##          Detection Rate : 0.007155
##    Detection Prevalence : 0.231150
##       Balanced Accuracy : 0.646440
##
##        'Positive' Class : Dead
##
```

Logistic Regression model

```
set.seed(197)
train.model.log.comp=train(x=dataset.x.tr.comp
                          ,y=dataset.y.tr.comp
                          ,method="LogitBoost"
                          ,trControl=ctrl
                          ,tuneGrid=expand.grid(nIter=seq(15,195,15))
                          ,importance=T
                          ,metric="ROC"
)
```

```
## Warning in nominalTrainWorkflow(x = x, y = y, wts = weights, info =
## trainInfo, : There were missing values in resampled performance measures.
```

Confusion matrix for Logistic model (complete cases)

```
pred.cm.log.comp=predict(train.model.log.comp,dataset.x.ts.comp)
cm.log.comp=confusionMatrix(pred.cm.log.comp,dataset.y.ts.comp,positive="Dead")
cm.log.comp
```

```
## Confusion Matrix and Statistics
##
##           Reference
## Prediction Alive Dead
##      Alive  1379    9
##      Dead    413   16
##
##                Accuracy : 0.7677
##                  95% CI : (0.7476, 0.787)
##     No Information Rate : 0.9862
##     P-Value [Acc > NIR] : 1
##
##                   Kappa : 0.0457
##  Mcnemar's Test P-Value : <2e-16
##
##             Sensitivity : 0.640000
##             Specificity : 0.769531
##          Pos Pred Value : 0.037296
##          Neg Pred Value : 0.993516
##              Prevalence : 0.013759
##          Detection Rate : 0.008806
##    Detection Prevalence : 0.236103
##       Balanced Accuracy : 0.704766
##
##        'Positive' Class : Dead
##
```

```
suppressWarnings({
set.seed(197)
train.model.svm.comp=train(x=dataset.x.tr.comp
                          ,y=dataset.y.tr.comp
                          ,method="svmLinear"
                          ,trControl=ctrl
                          ,tuneGrid=expand.grid(C=c(.0625,.125,.25,.5,1,2,4,8))
                          ,metric="ROC"
                          ,importance=T
                          ,probability=T

)
})
```

Confusion matrix for SVM model (complete cases)

```
pred.cm.svm.comp=predict(train.model.svm.comp,dataset.x.ts.comp)
cm.svm.comp=confusionMatrix(pred.cm.svm.comp,dataset.y.ts.comp,positive="Dead")
cm.svm.comp
```

```
## Confusion Matrix and Statistics
##
##           Reference
## Prediction Alive Dead
##      Alive  1413   13
##      Dead    379   12
##
##                Accuracy : 0.7843
##                  95% CI : (0.7646, 0.803)
##     No Information Rate : 0.9862
##     P-Value [Acc > NIR] : 1
##
##                   Kappa : 0.0327
##  Mcnemar's Test P-Value : <2e-16
##
##             Sensitivity : 0.480000
##             Specificity : 0.788504
##          Pos Pred Value : 0.030691
##          Neg Pred Value : 0.990884
##              Prevalence : 0.013759
##          Detection Rate : 0.006604
##    Detection Prevalence : 0.215190
##       Balanced Accuracy : 0.634252
##
##        'Positive' Class : Dead
##
```

Random Forest Model

```
set.seed(197)
train.model.rf.comp=train(x=dataset.x.tr.comp
                          ,y=dataset.y.tr.comp
                          ,method="rf"
                          ,trControl=ctrl.rf
                          ,tuneGrid=expand.grid(.mtry=c(1:tune.len))
                          ,importance=T
                          ,ntree=900
                          ,proximity=T
                          ,metric="ROC"
)
```

Confusion matrix for Random Forest model (complete cases)

```
pred.cm.rf.comp=predict(train.model.rf.comp,dataset.x.ts.comp)
cm.rf.comp=confusionMatrix(pred.cm.rf.comp,dataset.y.ts.comp,positive="Dead")
cm.rf.comp
```

```
## Confusion Matrix and Statistics
##
##           Reference
## Prediction Alive Dead
##      Alive  1575   12
##      Dead    217   13
##
##                Accuracy : 0.874
##                  95% CI : (0.8578, 0.8889)
##     No Information Rate : 0.9862
##     P-Value [Acc > NIR] : 1
##
##                   Kappa : 0.0791
##  Mcnemar's Test P-Value : <2e-16
##
##             Sensitivity : 0.520000
##             Specificity : 0.878906
##          Pos Pred Value : 0.056522
##          Neg Pred Value : 0.992439
##              Prevalence : 0.013759
##          Detection Rate : 0.007155
##    Detection Prevalence : 0.126582
##       Balanced Accuracy : 0.699453
##
##        'Positive' Class : Dead
##
```

Elastic Net model

```
set.seed(197)
train.model.net.comp=train(x=dataset.x.tr.comp
                          ,y=dataset.y.tr.comp
                          ,method="glmnet"
                          ,trControl=ctrl
                          ,tuneGrid=expand.grid(.alpha = seq(.05, 1, length = 15),
                          .lambda = c((1:5)/10))
                          ,metric="ROC"
                          ,family = "binomial"
)
```

Confusion matrix for Elastic Net model (complete cases)

```
pred.cm.net.comp=predict(train.model.net.comp,dataset.x.ts.comp)
cm.net.comp=confusionMatrix(pred.cm.net.comp,dataset.y.ts.comp,positive="Dead")
cm.net.comp
```

```
## Confusion Matrix and Statistics
##
##           Reference
## Prediction Alive Dead
##      Alive  1595   16
##      Dead    197    9
##
##                Accuracy : 0.8828
##                  95% CI : (0.8671, 0.8972)
##     No Information Rate : 0.9862
##     P-Value [Acc > NIR] : 1
##
##                   Kappa : 0.0547
##  Mcnemar's Test P-Value : <2e-16
##
##             Sensitivity : 0.360000
##             Specificity : 0.890067
##          Pos Pred Value : 0.043689
##          Neg Pred Value : 0.990068
##              Prevalence : 0.013759
##          Detection Rate : 0.004953
##    Detection Prevalence : 0.113374
##       Balanced Accuracy : 0.625033
##
##        'Positive' Class : Dead
##
```

6.2.2 AUC ROC curves for complete cases

```
pred.pls.comp=predict(train.model.pls.comp,dataset.x.ts.comp,type="prob")
pred.rf.comp=predict(train.model.rf.comp,dataset.x.ts.comp,type="prob")
pred.svm.comp=predict(train.model.svm.comp,dataset.x.ts.comp,type="prob")
pred.net.comp=predict(train.model.net.comp,dataset.x.ts.comp,type="prob")
pred.log.comp=predict(train.model.log.comp,dataset.x.ts.comp,type="prob")

roc(predictor=pred.rf.comp$Dead,response=dataset.y.ts.comp,levels=rev(levels(datas
et.y.ts.comp)),ci=T,plot=T,xlab="1 - Specificity")
```

```
##
## Call:
## roc.default(response = dataset.y.ts.comp, predictor = pred.rf.comp$Dead,     le
vels = rev(levels(dataset.y.ts.comp)), ci = T, plot = T,     xlab = "1 - Specifici
ty")
##
## Data: pred.rf.comp$Dead in 25 controls (dataset.y.ts.comp Dead) > 1792 cases (d
ataset.y.ts.comp Alive).
## Area under the curve: 0.7881
## 95% CI: 0.7078-0.8684 (DeLong)
```

```
rf.roc.comp=roc(predictor=pred.rf.comp$Dead,response=dataset.y.ts.comp,levels=rev(
levels(dataset.y.ts.comp)),ci=T,plot=T,add=T,col="#0000FF",
                xlim=c(0,1),main="ROC curves plotting performance of different mod
els")
pls.roc.comp=roc(predictor=pred.pls.comp$Dead,response=dataset.y.ts.comp,levels=re
v(levels(dataset.y.ts.comp)),ci=T,plot=T,add=T,col="#CD0000")
log.roc.comp=roc(predictor=pred.log.comp$Dead,response=dataset.y.ts.comp,levels=re
v(levels(dataset.y.ts.comp)),ci=T,plot=T,add=T,col="#000000")
svm.roc.comp=roc(predictor=pred.svm.comp$Dead,response=dataset.y.ts.comp,levels=re
v(levels(dataset.y.ts.comp)),ci=T,plot=T,add=T,col="#FFFF00")
net.roc.comp=roc(predictor=pred.net.comp$Dead,response=dataset.y.ts.comp,levels=re
v(levels(dataset.y.ts.comp)),ci=T,plot=T,add=T,col="#7CFC00")

legend('bottomright',c("AUC Results",
                      paste0("RF Model=",round(rf.roc.comp$auc[1],3)),
                      paste0("PLS-DA Model=",round(pls.roc.comp$auc[1],3)),
                      paste0("Logistic Model=",round(log.roc.comp$auc[1],3)),
                      paste0("Elastic-Net Model=",round(net.roc.comp$auc[1],3)),
                      paste0("Linear Kernel SVM Model=",round(svm.roc.comp$auc[1],
3))),
       lty = c(1,1,1,1,1,1),col=c('#FFFFFF','#0000FF','#CD0000','#000000',"#FFFF00
","#7CFC00"))
```

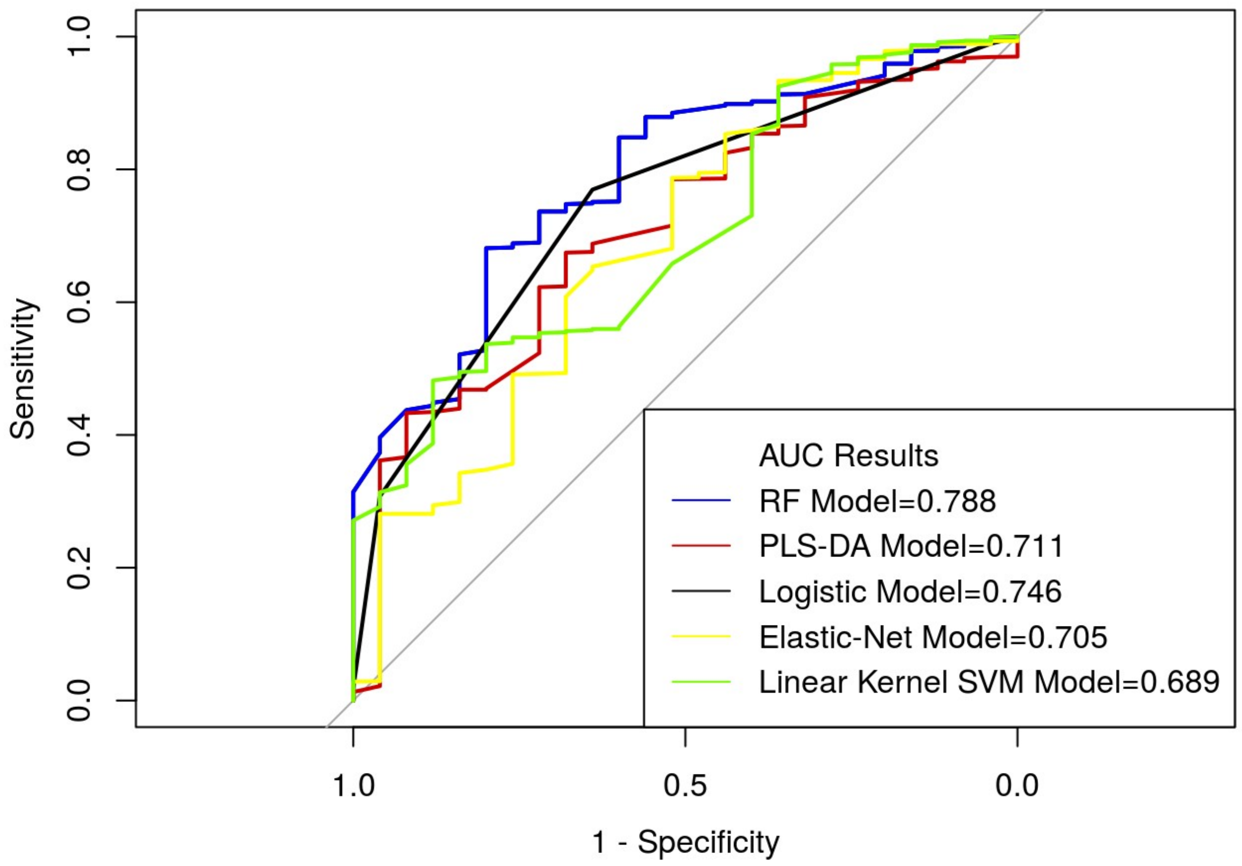


```
auc.comp.ci.df=data.frame("Model Type"=c("Logistic Model",
                                    "Random Forest",
                                    "PLS-DA",
                                    "Linear Kernel SVM",
                                    "Elastic-Net"),
                     "AUC"= c(round(log.roc.comp$auc[1],3),round(rf.roc.comp$auc[1
],3),round(pls.roc.comp$auc[1],3),
                              round(svm.roc.comp$auc[1],3),round(net.roc.comp$auc[
1],3)),
                     "95CI"=c(paste0(round(log.roc.comp$ci,3)[1],"-",round(log.roc
.comp$ci,3)[3]),
                              paste0(round(rf.roc.comp$ci,3)[1],"-",round(rf.roc.c
omp$ci,3)[3]),
                              paste0(round(pls.roc.comp$ci,3)[1],"-",round(pls.roc
.comp$ci,3)[3]),
                              paste0(round(svm.roc.comp$ci,3)[1],"-",round(svm.roc
.comp$ci,3)[3]),
                              paste0(round(net.roc.comp$ci,3)[1],"-",round(net.roc
.comp$ci,3)[3])))
kable(auc.comp.ci.df)
```

Model.Type	AUC	X95CI
Logistic Model	0.746	0.665-0.828
Random Forest	0.788	0.708-0.868
PLS-DA	0.711	0.615-0.808

Model.Type	AUC	X95CI
Linear Kernel SVM	0.689	0.574-0.803
Elastic-Net	0.705	0.611-0.798

```
stopCluster(cl)
stopImplicitCluster()
```
