# Supplementary figures and images for: An exploration of mortality risk factors in non-severe pneumonia in children using clinical data from Kenya
Source: BMC Med. 2017 Nov 13;15:201. doi: 10.1186/s12916-017-0963-9 (PMC5682642; doi:10.1186/s12916-017-0963-9)

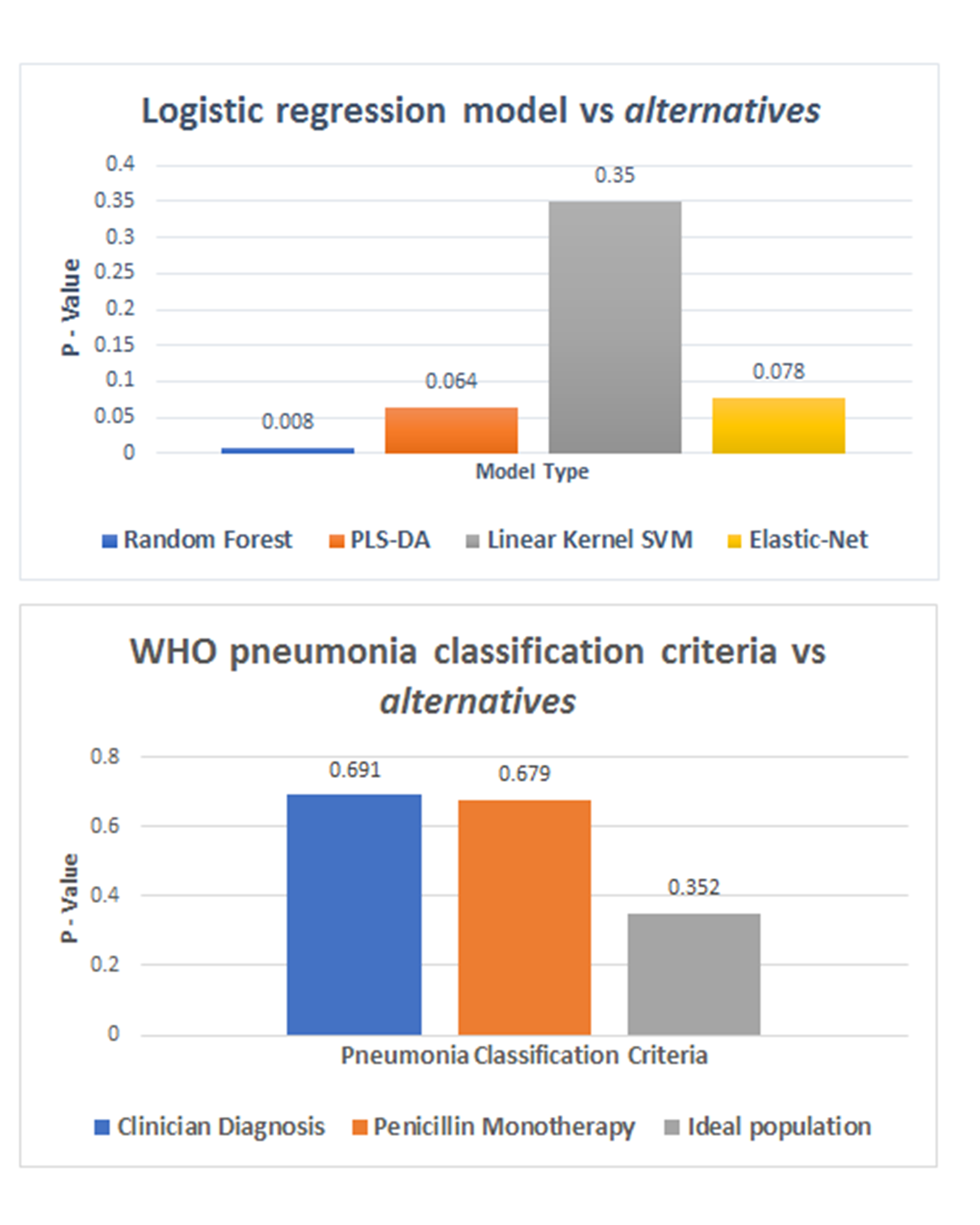

Supplement: Supplementary file 5 — Test for statistical significance of the difference between two AUC-ROC curves. AUC values provided in Fig. 2, 95% confidence interval values are provided in Additional file 2: Table S4. (TIF 7372 kb) [file 12916_2017_963_MOESM5_ESM.tif]
